# Bioreactor-grown exo- and endo-β-glucan from Malaysian *Ganoderma lucidum*: An *in vitro* and *in vivo* study for potential antidiabetic treatment

**DOI:** 10.3389/fbioe.2022.960320

**Published:** 2022-08-25

**Authors:** Nur Raihan Abdullah, Mohd Hamzah Mohd Nasir, Nur Hafizah Azizan, Wan Abd Al Qadr Imad Wan-Mohtar, Faez Sharif

**Affiliations:** ^1^ Department of Biotechnology, Kulliyyah of Science, International Islamic University Malaysia, Kuantan, Malaysia; ^2^ Functional Omics and Bioprocess Development Laboratory, Institute of Biological Sciences, Faculty of Science, Universiti Malaya, Kuala Lumpur, Malaysia

**Keywords:** Ganoderma lucidum, alpha-glucosidase, polysaccharide, embryo toxicity test, zebrafish

## Abstract

This study aims to identify the roles of exo-β-glucan (EPS-BG) and endo-β-glucan (ENS-BG) extracted from *Ganoderma lucidum* (GL) in inhibiting the alpha-glucosidase enzyme, a target mechanism for postprandial hyperglycaemia regulation. Upscale production of GL was carried out using a 10 L bioreactor. The zebrafish embryo toxicity test (ZFET) was carried out based on OECD guidelines. The hatching rate, survival rate, heart rate, morphological malformation, and teratogenic defects were observed and determined every 24 h from 0–120 h of post-exposure (hpe). For diabetes induction, adult zebrafish (3–4 months of age) were overfed and induced with three doses of 350 mg/kg streptozotocin (STZ) by intraperitoneal injection (IP) on three different days (days 1, 3, and 5). The oral sucrose tolerance test (OSTT) and anti-diabetic activity of EPS-BG and ENS-BG were evaluated (day 7) using the developed model (*n* = 15). This study showed that EPS is the most potent compound with the highest inhibitory effect toward the alpha-glucosidase enzyme with an IC_50_ value of 0.1575 mg/ml compared to ENS extracts (IC_50_ = 0.3479 mg/ml). Both EPS-BG and ENS-BG demonstrated a strong inhibition of alpha-glucosidase activity similar to the clinically approved alpha-glucosidase inhibitor, acarbose (IC_50_ = 0.8107 mg/ml). ENS-BG is non-toxic toward zebrafish embryos with LC_50_ of 0.92 mg/ml and showed no significant changes in ZE hatching and normal heart rate as compared to untreated embryos (161 beats/min). Teratogenic effects of ENS-BG (<1.0 mg/ml) on zebrafish embryonic development were not observed. The DM model of zebrafish was acquired after the third dose of STZ with a fasting BGL of 8.98 ± 0.28 mmol/L compared to the normal healthy group (4.23 ± 0.62 mmol/L). The BGL of DM zebrafish after 30 min treated with EPS-BG and ENS-BG showed a significant reduction (*p* < 0.0001). Both EPS-BG and ENS-BG significantly reduced DM zebrafish’s peak blood glucose and the area under the curve (AUC) in OSTT. Hence, EPS-BG and ENS-BG extracted from GL showed promising inhibition of the alpha-glucosidase enzyme and are considered non-toxic in ZE. Moreover, EPS-BG and ENS-BG reduced blood glucose levels and inhibited hyperglycemia in DM zebrafish.

## 1 Introduction

*Ganoderma lucidum (G. lucidum)* is one of the broadly utilized species in the biochemical and pharmaceutical fields. The research grew rapidly on the metabolite or complex produced, for example, ganoderic acid and polysaccharides for their medicinal convenience and was considered a “remedy that could resuscitate the dead” (Baby et al., 2015; Zhou et al., 2015; Ahmad et al., 2021; Du et al., 2021)*.* These days, Ganoderma has been utilized to prevent and treat numerous kinds of disorders and well-being items that are accepted to have anti-cancer properties, anti-aging, and are hostile to microbial or viral capacities. These products of *Ganoderma* are accessible, particularly in East Asia and the USA (Paterson, 2006; Seweryn et al., 2021). The number of publications that discussed the separation, bioactivity, and production of bioactive auxiliary metabolites of *G. lucidum* has expanded due to their extraordinary healthy benefit and their bottomless and interesting optional metabolites as a promising potential library for new medication disclosure.

Exopolysaccharide (EPS) is a high molecular weight polymer composed of sugar residues that are secreted by microorganisms into the surrounding environment as a response to environmental stress (Nicolaus et al., 2010; Poli et al., 2011). Endopolysaccharide (ENS), on the other hand, is the polymer that is produced inside the pellet mycelium during liquid fermentation and is believed to exhibit the same benefit as EPS. Significant attention has been made to discovering and developing new microbial EPS that possess novel industrial significance (Nicolaus et al., 2010). The vast functional properties of polysaccharides for pharmaceutical, food, and other industrial applications have led to the increased demand to produce this natural polymer. Moreover, a substantial amount of research has been reported to support the use of polysaccharides from *Ganoderma lucidum* in a broad range of applications other than medicine, including wastewater treatment, aquaculture, the food-biomass chain, and the production of protein-rich foods (Taufek et al., 2020).

Alpha-glucosidase inhibitors (AGI) such as acarbose and miglitol are particularly advantageous for reducing postprandial blood glucose levels by delaying the digestion of carbohydrates into glucose. Despite their effectiveness in diabetes management, these synthetic antidiabetic drugs were reported to have many side effects on diabetic patients’ health. The most often reported adverse effects of these medications are gastrointestinal problems. Acarbose lowers blood glucose by slowing down carbohydrate digestion. However, in the colon, the bacteria that degrade the undigested carbohydrates caause excessive gas formation, resulting in flatulence, diarrhea, and abdominal pain (Akmal and Wadhwa, 2021). Other than that, these synthetic drugs also pose a distinct adverse effect on diabetic patients, including hypersensitivity reactions, lactic acidosis, liver failure, acute pancreatitis, and weight gain (Chaudhury et al., 2017). The limitation of these drugs concerning the significant side effects on the patient has paved the way for the search for alternative solutions. Since EPS and ENS consist mainly of polysaccharides, their capability to act as alpha-glucosidase inhibitors is promising. Few studies have previously shown the inhibition activities of EPS toward the glucosidase enzyme. However, the EPS from previous studies were extracted mostly from plants and bacteria (Priatni et al., 2016; Sasikumar et al., 2017), and there is scarce literature that shows mushroom EPS and to the best of our knowledge, none of ENS as alpha-glucosidase inhibitors (Liu et al., 2018).

Zebrafish models to study human diseases have been established for human pathologies, including genetic disorders and diabetes (Intine et al., 2013; Salehpour et al., 2021). Compared to other vertebrate model organisms, the benefits of using zebrafish include high fecundity, short generation time, early adult transparency, reduced housing costs, and a variety of gene manipulation tools (Zang et al., 2018; Benchoula et al., 2019a; Mathews and Gustafsson, 2019; Salehpour et al., 2021). In addition, the zebrafish was successfully used for pharmaceutical discovery due to the extensive conservation of genetic pathways and cellular physiology among vertebrates and the ability to perform high-throughput drug screenings. There are several procedures available for inducing diabetes in the zebrafish model, including genetic and non-genetic approaches. Compared to other methods, genetic procedures may be more accurate in their ability to target specific genes and develop impairments that are more precise and specific in their outcomes (Heckler and Kroll, 2017; Middel et al., 2021; Salehpour et al., 2021). Non-genetic methods of diabetes induction, on the other hand, are preferable because they may be more commonly available, less expensive, or easier to perform (Krishnan and Rohner, 2019; Salehpour et al., 2021).

Reports have been made regarding the use of substances and extracts derived from GL as antidiabetic substances. GL has been shown previously to exert an antidiabetic effect; however, to date, an in-depth explanation of the mechanisms underlying its antidiabetic effect is still scarce. In this study, our focus was on the production of EPS-BG and ENS-BG from bioreactor-grown Malaysian *Ganoderma lucidum* for the regulation of postprandial hyperglycemia, focusing on inhibiting the alpha-glucosidase enzyme mechanism.

## 2 Materials and methods

### 2.1 Chemicals and reagents

The chemicals used for the experiments were all fresh and of analytical grade. Yeast extract (YE, Oxoid), magnesium sulfate (MgSO_4_, Bendosen), dipotassium phosphate (K_2_HPO_4_, Bendosen), mono-potassium phosphate (KH_2_PO_4_, Bendosen), ammonium chloride (NH_4_Cl, Bendosen), and D (+)- Glucose (R&M Chemicals). Alpha-glucosidase enzyme from *Saccharomyces cerevisiae* (Sigma-Aldrich), 4-Nitrophenyl glucopyranoside (pNPG, Sigma-Aldrich), heparin sodium salt (Solarbio), and Streptozotocin (Santa Cruz) were purchased in lyophilized powder form.

### 2.2 Microorganism

The mushroom mycelium of Malaysian *Ganoderma lucidum* strain QRS 5120 was obtained from the Functional Omics and Bioprocess Development Laboratory, University of Malaya. The media composition was 39 g/L of PDA powder for the plate subculture and seed culture, and the fermentation media were g/L glucose 30.0, yeast 1.0, KH_2_PO_4_ 0.5, K_2_HPO_4_ 0.5, MgSO_4_ 0.5, and NH_4_CL 4.0.

### 2.3 Liquid fermentation

#### 2.3.1 Fermentation in a shake flask

The method used for inoculum preparation involves two seed culture stages with both stages cultivated at 30°C with an initial pH of 4 and 100 rpm for 10 and 11 days, respectively (Wan Mohtar et al., 2016a). For the first seed culture, three mycelial agar squares cut from the PDA plate on day 10 were inoculated into a 100 ml medium (30 ml glucose, 50 ml mixed media, and 20 ml distilled water) in a 250 ml Erlenmeyer flask. For the second seed culture, 20% (v/v) of the mycelium from the first seed culture was taken and homogenized using a sterile hand blender for 10 s to produce additional growing hyphae tips. Then, the mycelium was transferred into a new medium in a 250 ml Erlenmeyer flask with 100 ml of the total working volume. The cultivation was carried out with pH 4, 100 rpm at 30°C (Incubation Shaker, Multitron Pro, INFORS HT, Switzerland).

#### 2.3.2 Upscale production using a bioreactor

The upscale production of *Ganoderma lucidum* was carried out using a 10 L Labfors bench-scale bioreactor (INFORS HT, 2012, Switzerland), which was constructed in accordance with the fungal heterotrophic stirred-tank bioreactor blueprint (Ahmad Usuldin et al., 2020). A total of 10% v/v of seed culture, from the shake flask fermentation, was inoculated into the media. In this experiment, the fermentation conditions were as follows: 30°C temperature, 1 VVM aeration rate, 100 rpm agitation speed, and an initial pH of 4. Then, EPS-BG and ENS-BG from the bioreactor-grown *Ganoderma lucidum* were isolated and identified as previously described (Abdullah et al., 2020).

### 2.4 Alpha-glucosidase enzyme inhibition assay

Serial dilutions of EPS-BG and ENS-BG were performed using a two-fold dilution factor (10 mg/ml, 5 mg/ml, 2.5 mg/ml, 1.25 mg/ml, and 0.625 mg/ml). The spectrophotometric method of Kazeem et al. (2013) with few modifications was used to determine the alpha-glucosidase inhibitory activity. In each 2 ml microcentrifuge tube, 50 μL of EPS-BG and ENS-BG solution was added. Then a solution of 100 μL of 1.0 U/mL alpha-glucosidase enzyme in 0.02 M sodium phosphate buffer, pH 6.9, was added to each tube, and the mixture was pre-incubated at 37°C for 10 min. Acarbose was used as a positive control and sodium phosphate buffer was used as a negative control. After incubation, 50 μL of 3.0 mM pNPG dissolved in buffer was mixed into the solution to start the reaction. The mixture reaction was stopped by adding 2 ml of 0.1 M Na_2_CO_3_ after incubating for 20 min at 37°C. The conversion rate of pNPG to p-nitrophenol was determined by the measurement of absorbance at 405 nm. The percentage of alpha-glucosidase inhibition was calculated as per the equation:

$$\mathrm{Percentage of inhibition (\%)}=\frac{\mathrm{ABS}\;\mathrm{negative}\;\mathrm{control}–\mathrm{ABS}\;\mathrm{sample}}{\mathrm{ABS}\;\mathrm{negative}\;\mathrm{control}}\times 100.$$

A dose-response curve was plotted using GraphPad Prism 8 to calculate the estimated IC_50_ value of crude EPS-BG, ENS-BG, and the positive control, acarbose, in this experiment.

#### 2.4.1 Enzyme kinetics of alpha-glucosidase

Kinetic tests were performed on EPS-BG and ENS-BG to assess the type of inhibition exerted on the alpha-glucosidase enzyme. The experiment was conducted according to the method by Kazeem et al. (2013). A constant dose of EPS-BG and ENS-BG (5 mg/ml) with the alpha-glucosidase enzyme was tested on the varied concentration of the substrate (0.094–3.0 mM of pNPG), as described in section 2.4, and the experiment was carried out in 96-well microplates. The measurement of p-nitrophenol was measured every 5 min for 35 min at 405 nm using a multifunctional microplate reader (Tecan Infinite 200 PRO). The Lineweaver–Burk plots were drawn using Prism software to determine the K_M_ (Michaelis constant) and V_max_ (maximum velocity) of the enzyme and the type of inhibition for the alpha-glucosidase enzyme.

### 2.5 Zebrafish embryo toxicity assay

#### 2.5.1 Ethics declaration

The use of zebrafish brood stocks was authorized by the Institutional Animal Care and Use Committee (IACUC), Faculty of Biotechnology and Biomolecular Sciences, Universiti Putra Malaysia.

#### 2.5.2 Zebrafish maintenance and breeding

A few pairs of adult zebrafish were placed in a breeding tank before the day of the breeding setup, where the female and male were separated by a separator. On the next day, the separator was removed, and the zebrafish embryos were collected from the egg collector (after 1 h of separator removal), washed, and incubated in embryo media, Danio-SprintM solution, for approximately 2 h. Dead or coagulated embryos were discarded, and healthy fertilized embryos were selected for use in the zebrafish embryo toxicity assay.

#### 2.5.3 Sample preparations for the toxicity test

Stock solutions (4 mg/ml) of ENS-BG were prepared by dissolving in the embryo media, Danio-SprintM. Sub-stocks were prepared in a 96-well microplate (200 µL) and diluted to six concentrations ranging from 0.06 to 2 mg/ml by serial dilution using embryo media in a 96-well microplate. Embryos, as in embryo media, were used as control (untreated).

#### 2.5.4 Zebrafish embryo toxicity test

The zebrafish embryo toxicity assay was carried out based on the Organization for Economic Cooperation and Development (OECD) guideline for the fish embryo toxicity (FET) test (OECD, 2013). Briefly, zebrafish embryos (one embryo/well) at 0 h of post-fertilization (0 hpf) were exposed to ENS-BG (200 µL) in 96-well microplates at seven different concentrations ranging from 0.01 to 10 mg/ml. The samples were tested with a total of 12 embryos per exposure group. The embryos exposed to the ENS-BG treatment were incubated at room temperature (25–28°C) for 5 days. The cumulative mortality and developmental malformations of embryos and larvae were observed and determined every 24 h from 0–120 h of post-exposure (hpe). The survival rate, hatching rate, heart rate, morphological malformation, and teratogenic defects were observed, and images/videos were captured/recorded using an inverted microscope attached to a digital camera. The heartbeat was counted from three selected embryos using a stopwatch for 1 min. Lethal endpoints were characterized by coagulation and no heartbeat. Developmental anomalies include pericardial edema, yolk sac edema, non-hatched, curved body, and bent tail. The mortality rate graph was used to obtain the lethal concentration 50 (LC_50_), a concentration of a chemical which in this experiment, ENS-BG, killed 50% of the zebrafish embryo at 120 hpf.

### 2.6 *In vivo* study of diabetes mellitus using zebrafish

#### 2.6.1 Ethical statement

The *in vivo* study of adult zebrafish was approved by the Institutional Animal Care and Use Committee (IACUC) of the International Islamic University Malaysia (IIUM) (Reference: IIUM/504/14/2/IACUC), and the study was carried out in accordance with the Malaysian Animal Welfare Act 2015 for the care and use of animals for scientific purposes.

#### 2.6.2 Zebrafish husbandry and experimental design

A total of 108 male and female adult wild-type zebrafish (*Danio rerio*) aged 3–5 months were used throughout the study (75 for the experiment and 33 for the pilot study). The zebrafish were purchased from a local pet store in Kuantan and acclimated in standard laboratory conditions using a photoperiod of 14 h light and 10 h dark at a temperature of 26 ± 2°C for 10 days. During the acclimation period, the fish were treated with an antifungal (API MelaFix) and an antibacterial (API MelaFix) to avoid any disease transfer into the facility. The experiment was carried out at the IIUM Central Research and Animal Facility (CREAM), with each fish placed in a group of 15 in an 8 L acrylic fish tank filled with filtered facility water and aerated with air bubbles and biofilter stones. The zebrafish were fed with a commercial fish diet Otohime B2 (Marubeni Nisshin Feed, Japan).

For the experimental design, the zebrafish were randomly divided into a total of five groups (*n* = 15), including the control group for normal healthy zebrafish and the control group for induced diabetic zebrafish without any treatment. The treatment group for induced diabetic zebrafish consisted of EPS-BG, ENS-BG, and one with the treatment of the current standard drug, acarbose. All groups were subjected to the oral sucrose tolerance test (OSTT) at the end of the study.

#### 2.6.3 Pain management (anesthesia/euthanasia method)

The fish were anaesthetized using ice water (gradually from 12–10°C) for 30 s. After injection, the fish will be immediately transferred back to the recovery tank (18°C to reduce the pain after injection) (Kinkel et al., 2010; Zang et al., 2017). According to Matthews and Varga (2012), using cooling or hypothermia as a method of anesthesia is more effective and has been used as a simple method of short-term immobilization. For our project, we anesthetize the fish to take the blood sample and inject the STZ. After injection, the zebrafish were immediately transferred back to the warm-water (about 28°C) tank for recovery. To relieve wound healing and prevent any infection at the injection site, antibacterial (API Melafix) was added to the recovery tanks.

Methods for euthanasia of adult zebrafish were carried out by overdosing them with MS-222 euthanizing solution with a concentration of 300 mg/L (Matthews and Varga, 2012). The zebrafish were exposed to the solution for at least 10 min following cessation of opercular movement before removing the fish from the euthanizing solution.

#### 2.6.4 Weight measurement

The zebrafish were moved to a separate tank and labeled appropriately after each one was relocated. The beaker filled with 1/4 of the facility water was placed on the balance and the balance was tared. A fish net was employed, and the surplus water was wiped away with the towel that had been prepared earlier. The zebrafish was moved to a tared weighing beaker, where the weight was measured and noted down.

#### 2.6.5 Induction of diabetes using overfed and streptozotocin injection method

The zebrafish were overfed, according to Zang et al. (2017), with few modifications. For the induced diabetic group (DM), the zebrafish were fed with 120 mg of fish feed, Otohime B2, per day (8% more than the normal diet), divided over four daily feedings. The zebrafish were fed for a total of 15 min at each mealtime. The excess feed was removed after 30 min to avoid ammonia poisoning caused by degraded food that had not been consumed. The overfeeding method was started 10 days before the induction of streptozotocin (STZ). This study used STZ from Sigma-Aldrich to induce diabetes in zebrafish and followed the method done by Intine et al. (2013) and Abbas et al. (2017) with few modifications. A total of 6 mg of streptozotocin (STZ) was added to 2 ml of 0.09% sodium chloride to prepare a 0.3% solution of streptozotocin solution in a microcentrifuge tube covered with aluminum foil and immediately placed on ice, and saline solution was used as control.

**FIGURE 1:**
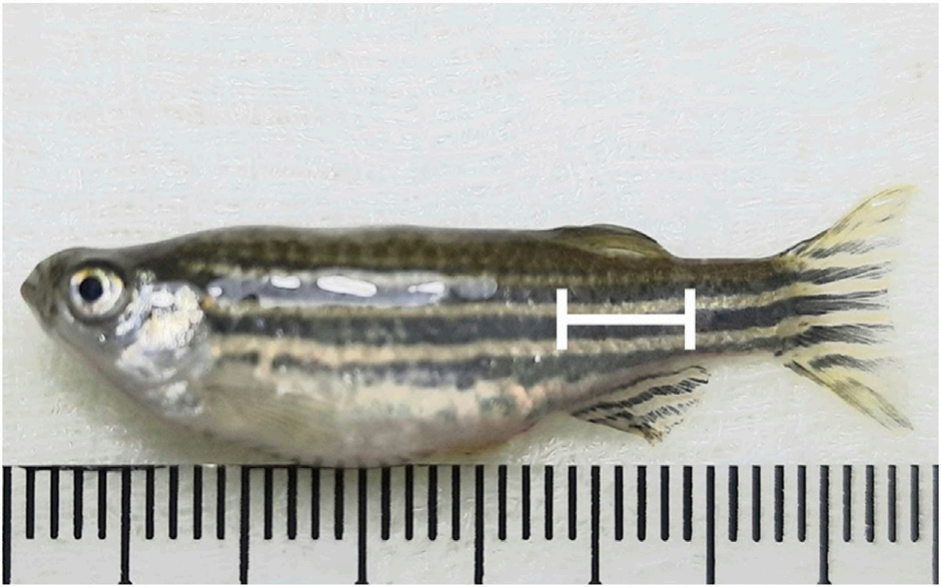
Intraperitoneal injection of STZ into zebrafish.

A TERUMO insulin syringe with needle size 31G was used and filled with STZ or control solutions. Fish were anesthetized individually by placing the fish in cold water. Anesthesia is achieved when the swimming motion of the fish ceased. Anesthetized zebrafish were placed on the cold sponge, and the head was covered with cold gauze for the injection of STZ or control solution. The solution was injected into the peritoneal cavity (Figure 1) of the fish by inserting the needle past the bevel into the posterior side of the fish’s ventral peritoneum (Intine et al., 2013). A total of 350 mg/kg of STZ was injected into each zebrafish.

After induction, the injection site was pressed for a while to minimize the injury caused by the needle, and the zebrafish were placed in the recovery water tank and monitored for normal swimming activity. Then, the zebrafish were transferred into a normal living tank where the temperature was maintained at the range of 24°C–28°C. This reduced temperature is critical for the efficient induction of hyperglycemia, and it can be detected within 24 h of the injection (Intine et al., 2013).

A repeated dose of STZ injection was carried out to induce a prolonged state of hyperglycemia. The induction of STZ was carried out on days 1, 3, and 5. On day 7, the treatments were administered after the confirmation of the induced diabetic zebrafish model. The zebrafish were considered to have been in a prolonged state of hyperglycemia and exhibited diabetic complications of retinopathy, nephropathy, and also impaired fin regeneration (Intine et al., 2013).

#### 2.6.6 Repeated blood collection for a blood glucose reading

Several protocols have been developed for blood collection, even though the small size of adult zebrafish (3–4 cm) makes it challenging for the blood collection process, including the methods that require the animal to be sacrificed (Zang et al., 2018). However, Zang et al. (2015) developed a method that makes it possible to collect blood repeatedly in the same individual adult zebrafish, and the blood glucose level can be measured by using glucometers (Zang et al., 2015, 2018).

**FIGURE 2:**
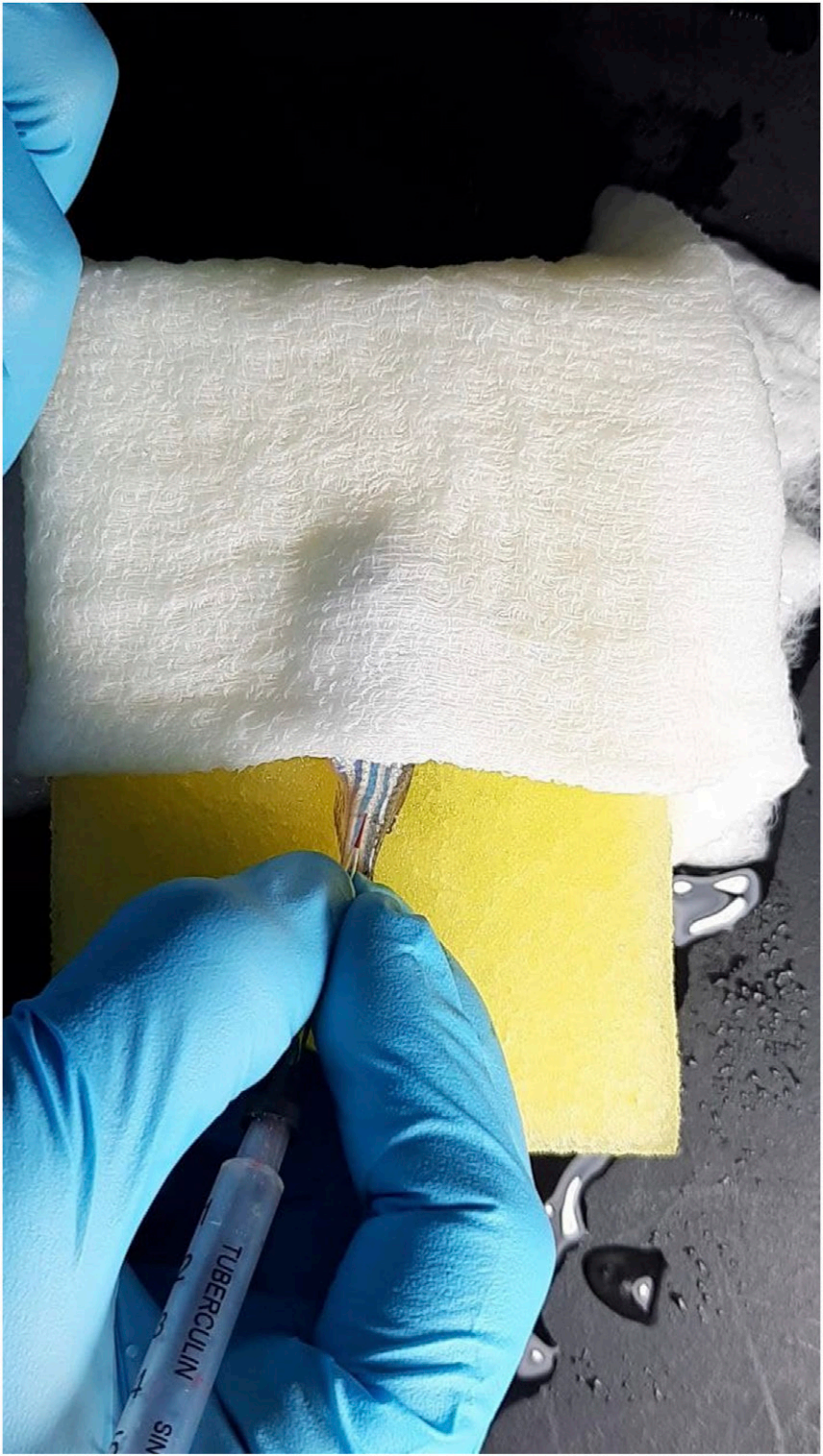
Collection of zebrafish blood using a Z-fish needle.

The fish were anesthetized, as mentioned in 2.6.3, and a heparinized capillary needle was used for blood collection to avoid blood coagulation. The method used for blood collection followed the one by Zang et al. (2015), where the site for blood collection is in the region of the dorsal aorta, along the body axis, and posterior to the anus (Figure 1). The micro-capillary needle was inserted at a 30–45° angle into the blood collection site (Figure 2), and the blood slowly rose without suction (arterial blood pressure). The suction was stopped after ∼2 µL volume of blood was collected. The needle was pulled, and the puncture site was gently squeezed using a hand to stop any bleeding. The fish will be immediately transferred back to a recovery tank after the bleeding is stopped. The collected blood from the needle was expelled and put onto a clean area of a piece of parafilm. A glucometer (Accu-Chek Instant S meter) was used to measure the blood glucose level of the zebrafish.

#### 2.6.7 Oral administration of EPS-BG and ENS-BG using the force-feed method

DM zebrafish were administered with acarbose, EPS-BG, and ENS-BG dissolved in saline to a final concentration of 5 mg/ml using the force-feed method. Both normal control and DM control groups were treated with saline solution. The blood sample was collected after 30 min of treatment administration for the measurement of the blood glucose level.

#### 2.6.8 Oral sucrose tolerance test

A sucrose solution was administered at a dose of 1,250 mg/kg zebrafish weight using a small tip micropipette. The micropipette was inserted gently into the mouth of the anesthetized zebrafish for the OSTT. Then, the zebrafish were allowed to recover for 30, 60, and 120 min after dosing in the water system, and at each time point, the blood glucose level was determined using the collected blood. The value of the area under the curve (AUC) for the blood glucose level was determined with prism software using the trapezoid rule.

### 2.7 Statistical analysis

All graphs and data were generated by using GraphPad Prism version 8.0 (GraphPad Software, Inc.). The data were reported as mean ± SEM. The significant differences were determined using one-way analysis of variance (ANOVA) and a post hoc test (Dunnett’s Multiple Comparison and Tukey’s test). The value of *p* < 0.05 was considered to indicate statistical significance. The absence of an error bar indicates that the symbol’s size exceeds the error value.

## 3 Result and discussion

### 3.1 *In vitro* alpha-glucosidase inhibitory assay

Alpha-glucosidase is an intestinal brush border enzyme that plays a role in the hydrolysis of oligosaccharides into glucose and the breakdown of sucrose into glucose and fructose (Lankatillake et al., 2019; Ji et al., 2021). By delaying carbohydrate digestion and reducing the absorption of monosaccharides, the inhibitor can play a vital role in suppressing the postprandial blood glucose levels of diabetic patients and maintaining normal blood glucose levels (Lankatillake et al., 2019; Zhang et al., 2020). In this study, the alpha-glucosidase inhibitory activities of EPS-BG and ENS-BG were evaluated by *in vitro* assays and compared to acarbose as a positive control.

Table 1 shows the percentage inhibition of acarbose, EPS-BG, and ENS-BG toward alpha-glucosidase enzyme at five different concentrations ranging from 0.625–10 mg/ml. Both EPS-BG and ENS-BG exhibit significant inhibitory activity, with the percentage of inhibition exceeding more than 80%. The data depicted an increasing trend, indicating that the inhibitory activity of the sample increases as the sample concentration increases. When compared to ENS-BG, the percentage of EPS-BG enzyme inhibition was statistically significant with a higher inhibition value. At the lowest concentration, 0.625 mg/ml, the inhibition percentage for EPS-BG and ENS-BG was 94.71% and 89.76% inhibition, respectively, while, at the highest concentration, 10.0 mg/ml, the inhibition percentages were 98.73% for EPS-BG and 96.98% for ENS-BG.

**TABLE 1:** Estimated IC_50_ value and the percentage of alpha-glucosidase enzyme inhibition activity by EPS-BG, ENS-BG, and acarbose.

Sample	Sample concentration (mg/ml)	Percentage of inhibition (%)	IC_50_ value (mg/ml)
EPS-BG	0.625	94.71 ± 0.21	0.1575
	1.250	95.92 ± 0.09	
	2.500	96.03 ± 0.06	
	5.000	97.88 ± 0.11	
	10.000	98.73 ± 0.12	
ENS-BG	0.625	89.760 ± 0.13	0.3479
	1.250	91.650 ± 0.06	
	2.500	93.270 ± 0.08	
	5.000	95.830 ± 0.10	
	10.000	96.980 ± 0.16	
Acarbose	0.625	15.250 ± 0.16	0.8107
	1.250	26.974 ± 0.16	
	2.500	28.728 ± 0.09	
	5.000	86.842 ± 0.15	
	10.000	95.614 ± 0.11	

The inhibitory activity of EPS-BG and ENS-BG against alpha-glucosidase enzyme was then compared to the standard positive control acarbose. The inhibition activities of acarbose at lower concentrations (0.625–2.5 mg/ml) were lower, with only 15–30% of the inhibition activity compared to EPS-BG (94–96%) and ENS-BG (89–93%). Nevertheless, for all tested groups, the highest concentration tested, 10.0 mg/ml, demonstrated the greatest inhibition activity for each acarbose (95.61%), EPS-BG (98.73%), and ENS-BG (96.98%).

The estimated 50% inhibitory concentration (IC_50_) values for acarbose, EPS-BG, and ENS-BG are listed in Table 1. IC_50_ is the concentration of the compound required to obtain 50% inhibition of alpha-glucosidase activity under the assay conditions. The log concentration graphs of acarbose, EPS-BG, and ENS-BG were plotted against their respective percentage inhibitory effects. According to the estimated value, EPS-BG has the lowest IC_50_, 0.1575 mg/ml, when compared to ENS-BG (0.3479 mg/ml) and acarbose (0.8107 mg/ml). Generally, a lower IC_50_ value indicates better inhibitory activity, as it suggests less sample required to inhibit the enzyme (Ji et al., 2021).

The IC_50_ value of *G. lucidum* extracts on the alpha-glucosidase enzyme inhibition activity in several studies varied*.* According to Satria et al. (2019), the sample harvested from *G. lucidum* fruiting bodies 31–34 weeks after inoculation was the strongest inhibitor, with an IC_50_ value of 27 ± 1.4 μg/ml, and a study by Chen et al. (2019) reported an IC_50_ value of 31.82 ± 4.30 μg/ml against alpha-glucosidase activity. Moreover, the inhibition activity of *G. lucidum* extracts against the alpha-glucosidase enzyme from the rat’s small intestinal mucosa was carried out by Chen et al. (2017) with an IC_50_ value of 0.6–91.2 μM. The IC_50_ value varies among the studies that have been published because the samples used and the extraction methods employed were all different. However, all these findings supported the roles of the sample used in the current study (EPS-BG and ENS-BG) as one of the natural potent alpha-glucosidase inhibitors and can play a role in controlling blood glucose levels.

#### 3.1.1 Kinetics of alpha-glucosidase enzyme inhibition

**FIGURE 3:**
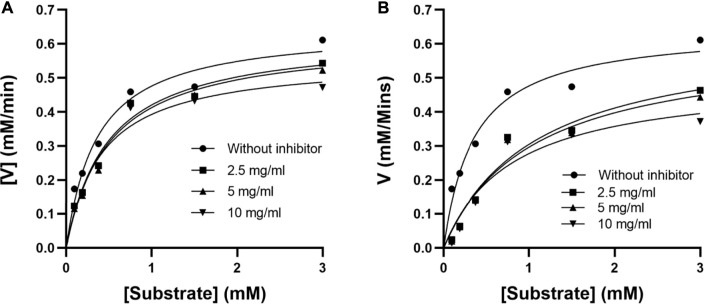
Michaelis–Menten plots of **(A)** EPS-BG and **(B)** ENS-BG.

**FIGURE 4:**
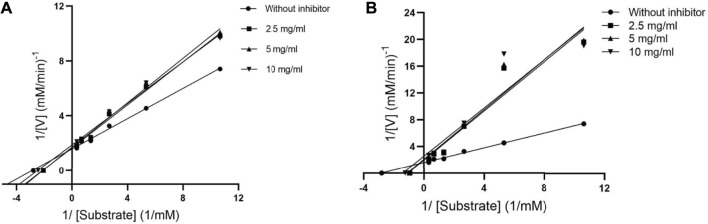
Lineweaver–Burk plots of **(A)** EPS-BG and **(B)** ENS-BG.

The mode of alpha-glucosidase inhibition by EPS-BG and ENS-BG was determined by Michaelis–Menten kinetics and Lineweaver–Burk plots. Figure 3 and Figure 4 show the kinetics of the inhibitory mode of alpha-glucosidase enzymes exerted by EPS-BG and ENS-BG. The Lineweaver–Burk plots reveal that both EPS-BG and ENS-BG inhibited alpha-glucosidase in a mixed-type manner. A reversible mixed type of inhibition was indicated by the data points on the Lineweaver–Burk of both EPS-BG and ENS-BG intersect in the second quadrant (Luthra et al., 2017) (Figure 4), and altered the values of both the Michaelis–Menten constant (*K*_m_) and maximum enzyme velocity (*V*_max_) (Mohammed et al., 2017) (Table 2). The data show that the *V*_max_ values of EPS-BG and ENS-BG decreased as the concentration of the compounds increased. A mixed inhibitor (EPS-BG and ENS-BG) changes the K_m_ and V_max_ values of the enzyme by interfering with the binding of substrates to the active site (Zaharudin et al., 2019).

**TABLE 2:** Kinetic parameters of alpha-glucosidase inhibition by EPS-BG and ENS-BG from *G. lucidum* mycelial extracts.

Inhibitor	Inhibitor concentration	*K*_m_ (mM)	*V*_max_ (mM/min^−1^)
EPS-BG	0.000	0.357	0.647
	2.500	1.074	0.633
	5.000	1.031	0.603
	10.000	0.807	0.502
ENS-BG	0.000	0.357	0.647
	2.500	0.483	0.625
	5.000	0.490	0.616
	10.000	0.410	0.554

### 3.2 Zebrafish embryo toxicity assay

#### 3.2.1 Zebrafish embryo survival rate after ENS-BG exposure

**FIGURE 5:**
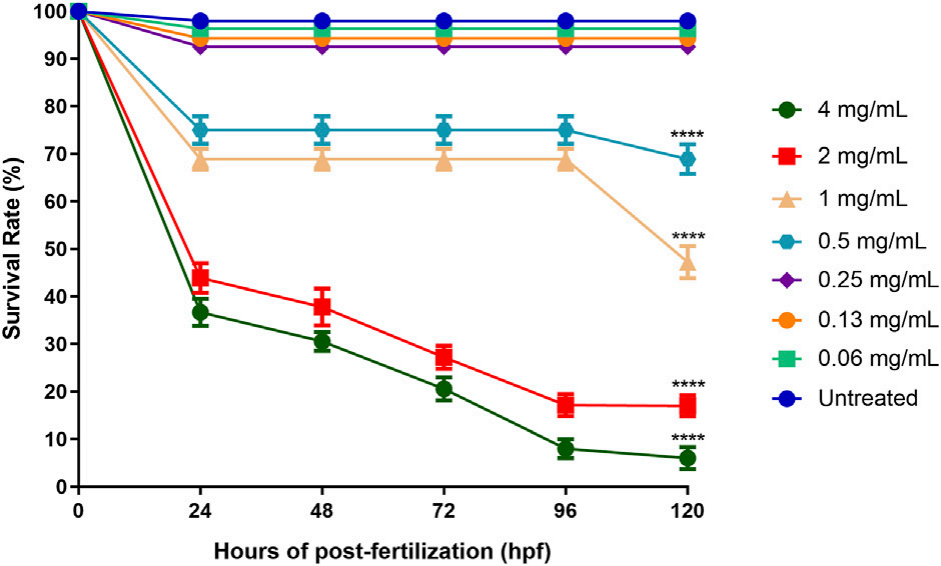
Survival rate of zebrafish embryos at 0–120 h after exposure to ENS-BG at concentrations of 0.06–4 mg/ml. Embryo survival at a concentration of 4.0 mg/ml after 96 h of post-fertilization (hpf) dropped to 10%. One-way analysis of variance (ANOVA) was used to carry out the significant differences with a post-hoc test using Dunnett’s Multiple Comparison. The significant difference was considered at *p ˂ 0.05, **p < 0.01, and ***p < 0.001 between the means of the treated group as compared to zebrafish embryos in embryo media only (untreated).

The survival rate of the embryos of the zebrafish for each treated (ENS-BG concentration ranging from 0.06–4 mg/ml) and untreated (control, Danio-SprintM solution) group was observed with a baseline (100% survival rate) of 12 embryos per exposure group. According to the standard, zebrafish embryos normally hatch between 48 and 72 h post-fertilization (hpf). As a result, the rate of embryo survivability (before hatching) and larvae (post-hatch) treated with ENS-BG was determined for a maximum of 5 days (120 h) with every 24 hpf observation. Figure 5 shows that at 0 hpf, all the treatment groups started with a 100% survival rate before the rate declined for every next 24 hpf. The embryos of the untreated group (control, Danio-SprintM solution) survived up to 5 days (120 hpf). The survival rate dropped slightly (92.56%) when exposed to ENS-BG at the concentration ranges of 0.06–0.24 mg/ml. At 0.5 and 1.0 mg/ml of ENS-BG concentrations, the survival rate observed at 24 to 96 hpf was 75% and 68.89% respectively, before it dropped to 68.89 % and 47.22% at 120 hpf. The survival rate dropped significantly at 2 and 4 mg/ml of ENS-BG concentration at 24 hpf from 100% to 43.89% and 36.67%, respectively. At 120 hpf, only 6% of the embryos were able to survive after being treated with the highest concentration (4 mg/ml) and a 17% survival rate for 2 mg/ml of ENS-BG.

#### 3.2.2 Zebrafish embryo’s mortality rate after ENS-BG exposure

**FIGURE 6:**
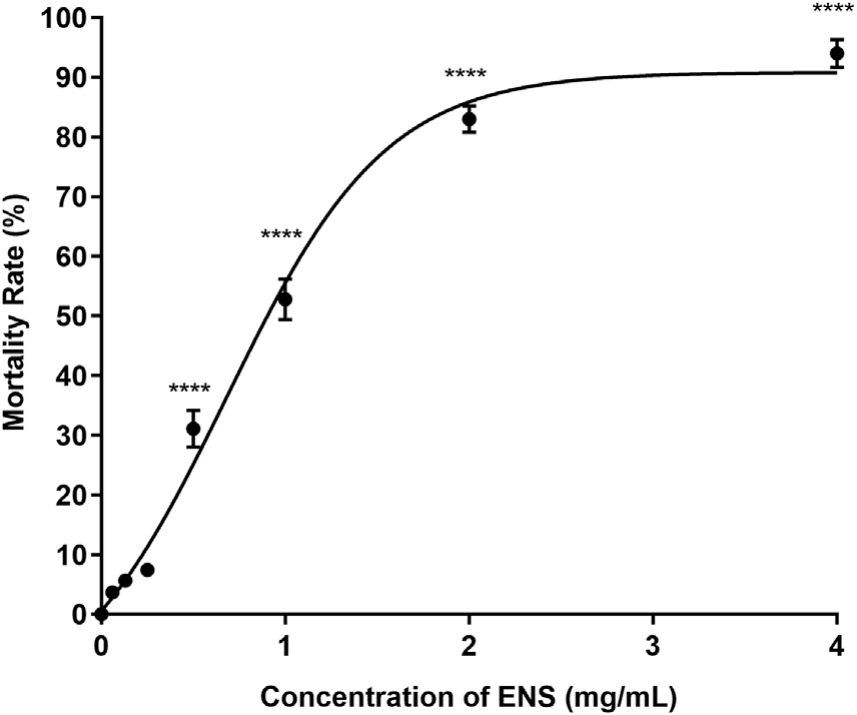
Effect of ENS-BG at concentrations of 0.06–4 mg/ml on zebrafish embryos’ mortality rate after 120 h of post-fertilization (hpf). One-way analysis of variance (ANOVA) was used to carry out the significant differences with a post-hoc test using Dunnett’s Multiple Comparison. The significant difference was considered at *p ˂ 0.05, **p < 0.01, and ***p < 0.001 between the means of the treated group as compared to zebrafish embryos in embryo media only (untreated).

The lethal effect of ENS-BG on the embryo varied depending on the dose and the period of exposure. Figure 6 shows the effect of ENS-BG at the concentrations of 0.06–4 mg/ml on the zebrafish embryo’s mortality rate after 120 h of post-fertilization (hpf). Overall, the mortality rate increased as the dose of ENS-BG increased. More than 50% mortality rate was reported for the embryo treated with 1.0–4.0 mg/ml ENS-BG, where 4 mg/ml ENS-BG showed the highest dead embryos (94%) and lowest survival rate. However, ENS-BG at a concentration less than 1 mg/ml yielded a high survival rate (92%) with only an 8% mortality rate. Similar results were reported by Taufek et al. (2020) and Dulay et al. (2012), where the concentration of EPS less than 2 mg/ml and the concentration of *G. lucidum* extract less than 1% showed a >90% survival rate. These indicated that the lethal effect of ENS-BG was dose- and time-dependent. It was determined that by applying the mortality rate graph, the LC_50_ value of ENS-BG toward zebrafish embryos, calculated with the help of PRISM 8 software, was 0.920 mg/ml.

#### 3.2.3 Zebrafish embryo’s hatching rate after ENS-BG exposure

**FIGURE 7:**
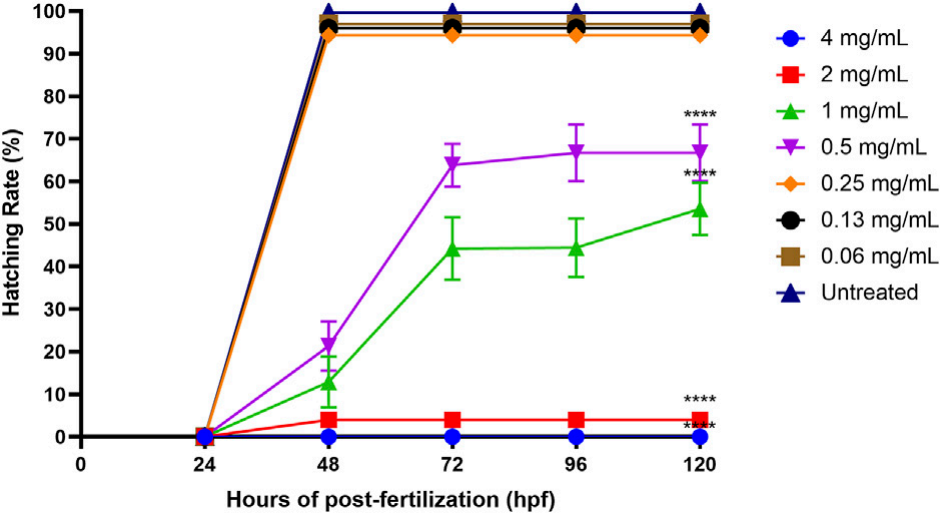
Hatching rate of zebrafish embryos at 0–120 h of post-exposure with ENS-BG at concentrations of 0.06–4 mg/ml. A low hatching rate (<10%) was observed at concentrations >2.0 mg/ml due to the high mortality rate. One-way analysis of variance (ANOVA) was used to carry out the significant differences with a post-hoc test using Dunnett’s Multiple Comparison. The significant difference was considered at ****p < 0.0001 between the means of the treated group as compared to zebrafish embryos in embryo media only (untreated).

The standard time for a zebrafish embryo to normally hatch is between 48 and 72 hpf, and the hatching rate might be affected by different treatment concentrations. Figure 7 shows that the hatching rates of zebrafish embryos decreased with the increasing concentrations of ENS-BG (>0.5 mg/ml). The hatching rate of the untreated group (control) was 99.67% at 48 hpf. There was no significant difference detected in the hatching rate of embryos treated with 0.06–0.25 mg/ml of ENS-BG (>94%) at 48 hpf compared to the control group. At 0.5 and 1.0 mg/ml of ENS-BG concentrations, the hatching rate observed at 48 hpf was 21% and 12% and increased to 66 % and 53% at 120 hpf, respectively. The hatching rate was extremely low at 2 and 4 mg/ml of ENS-BG concentration (<4%) due to the high mortality rate.

#### 3.2.4 Zebrafish embryo’s heart rate after ENS-BG exposure

**FIGURE 8:**
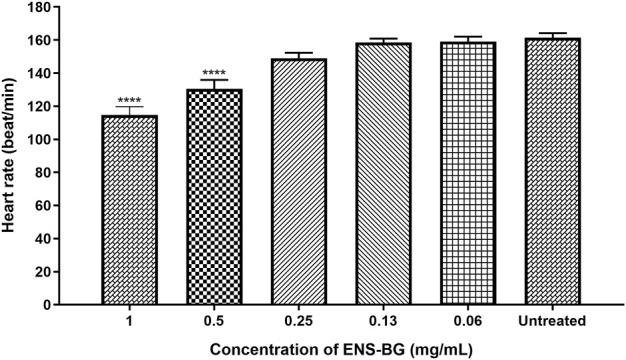
Effect of ENS-BG at concentrations of 0.06–4 mg/ml on the heart rate of zebrafish embryos at 96 hpf. No data at concentrations >2.0 mg/ml due to death. *p < 0.05 significantly different from the untreated group (zebrafish embryos in media only). The heart rate is presented as mean ± standard error of the mean (S.E.M) from three different embryos. One-way analysis of variance (ANOVA) was used to carry out the significant differences with a post-hoc test using Dunnett’s Multiple Comparison. The significant difference was considered at ****p < 0.0001 between the means of the treated group as compared to zebrafish embryos in embryo media only (untreated).

According to OECD guidelines, the heartbeat is visible only after 48 hpf in a normal developing zebrafish embryo at 26 ± 1°C. The average heart rate of zebrafish embryos was found to be 120–180 beats per minute (bpm), which was considered close to the rate found in humans (Bakkers, 2011). Figure 8 shows the heart rates of zebrafish embryos at 96 h post-fertilization (hpf) after being exposed to ENS-BG. The heart rate of zebrafish embryos for the untreated group (control) at 96 hpf was 161.3 ± 2.906 bpm. There was no significant difference detected in the heart rate of embryos treated with 0.06–0.25 mg/ml of ENS-BG at 96 hpf as compared to the control group. Nevertheless, at 0.5 and 1.0 mg/ml concentrations, the heart rate observed at 96 hpf was significantly reduced, 130.4 ± 5.414 and 114.7 ± 5.044 bpm, respectively. The heart rate at 2 and 4 mg/ml of ENS-BG concentration was not determined because of the very low survival rate at 96 hpf.

#### 3.2.5 Morphology and development of the larvae and zebrafish embryos after ENS-BG exposure

**FIGURE 9:**
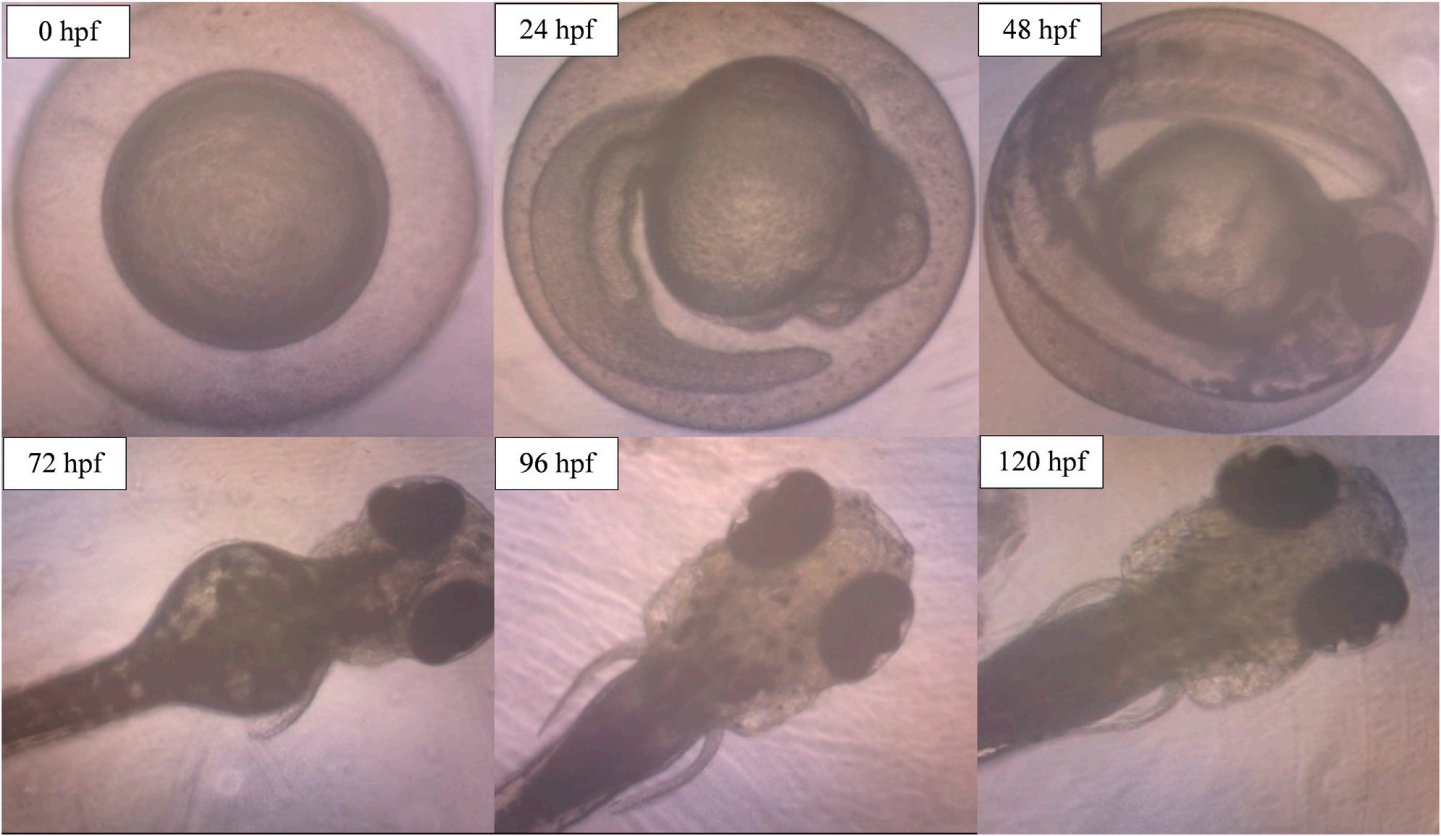
Image of zebrafish embryo and larvae development after treatment with ENS-BG at a concentration of 0.5 mg/ml. Images were captured using an inverted microscope at 100X (0–48 hpf) and ×40 magnification (72–120 hpf).

The potential occurrence of morphological and physiological abnormalities of zebrafish embryos to larvae after the exposure to ENS-BG was observed and measured at 0–120 hpf. Figure 9 shows the normal development of zebrafish embryos to becoming larvae without any teratogenic defects at 0.5 mg/ml concentration of ENS-BG. At 24 hpf, the embryo demonstrated development during the segmentation period which is indicated by the formation of somites and the appearance of the tailbud followed by the pharyngula period at 48 hpf where pigment cells and the eye are now present and well shown. The normal hatching periods are between 48 and 72 hpf, and the figure presents the hatched embryo at 72 hpf and the larval development up to 120 hpf. The protruding mouth and development of the fins were observed.

**FIGURE 10:**
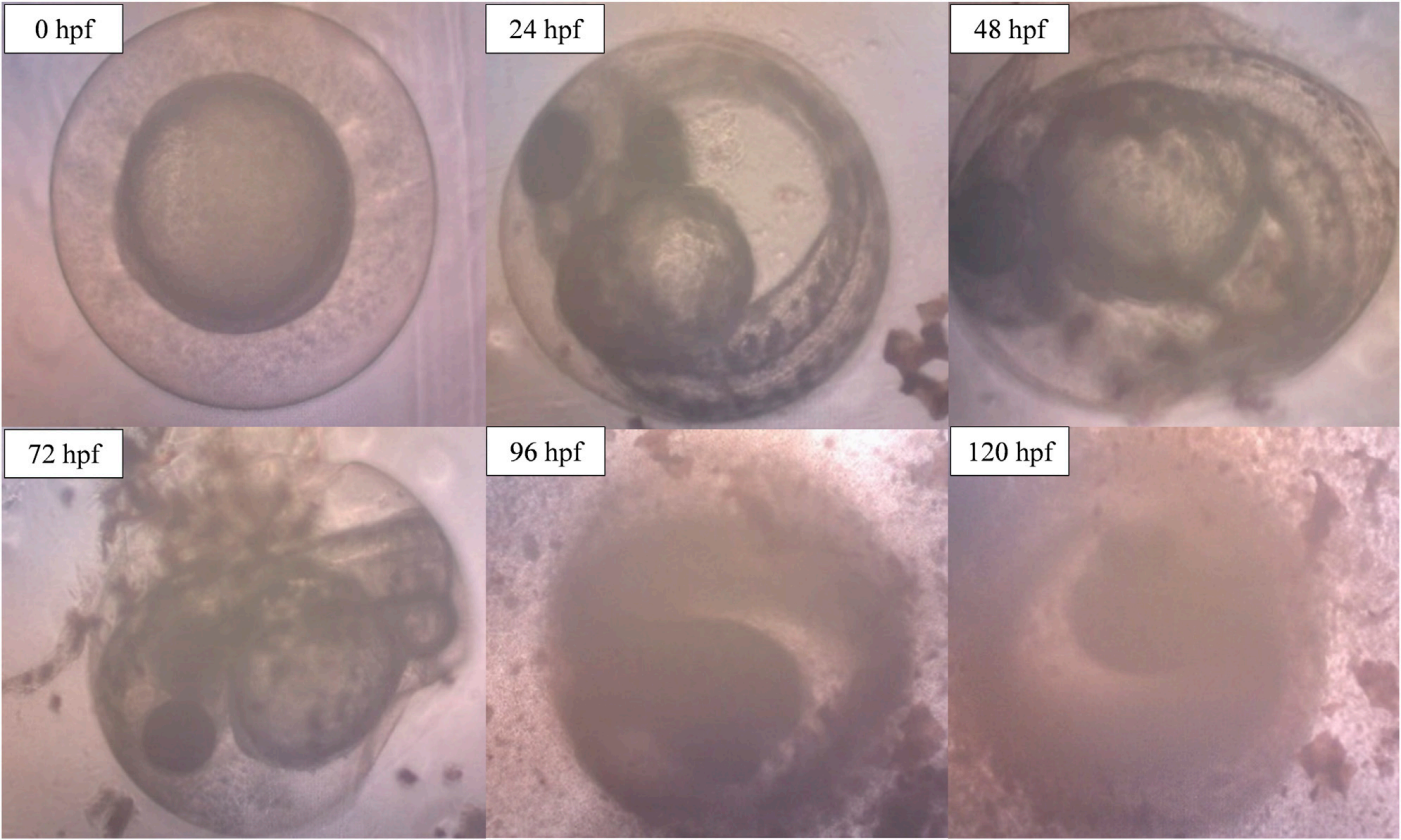
Image of zebrafish embryo and larvae development after treatment with ENS-BG at a concentration of 4.0 mg/ml. Images were captured using an inverted microscope at 100X (0–48 hpf) and ×40 magnification (72–120 hpf).

However, when the concentration of ENS-BG increased to 4.0 mg/ml, defects and abnormalities were observed (Figure 10). Tail malformation was observed with delayed and failure of hatching. The embryo started to coagulate, indicating a teratogenic defect followed by the decomposition of the dead embryo at 120 hpf.

#### 3.2.6 Non-toxicity assessment of ENS-BG from the mushroom *Ganoderma lucidum*

The zebrafish embryos were used to study the toxicity of ENS-BG extracted from *Ganoderma lucidum* QRS 5120 mycelium. This test is critical in determining whether or not the sample being used is safe before proceeding to analysis using adult zebrafish for the *in vivo* study. The model of the toxicity test using the embryo zebrafish offers several advantages, including fast embryonic development and optical transparency of embryos which allow direct observation of phenotypic changes (Taufek et al., 2020; Bauer et al., 2021).

The current study exposed the zebrafish embryos to various concentrations of ENS-BG (0.06–4.0 mg/ml), and the result suggested that ENS-BG is non-toxic and safe. Overall, ENS-BG at concentrations <0.5 mg/ml did not cause any delay in the hatching of zebrafish embryos with a high survival rate of >92%. Moreover, no teratogenic defects were observed during zebrafish embryonic development (0–120 hpf), and the heart rate was normal as the ENS-BG treatment did not have a significant effect when compared to the untreated embryo. However, there were visible teratogenic defects in the development of zebrafish embryos at concentrations of 4.0 mg/ml.

Based on the assay, ENS-BG was considered practically non-toxic with LC_50_ 0.920 mg/ml. A previous study by Taufek et al. (2020) found that the mycelial biomass (MB) and exopolysaccharide (EPS) extracted from the same strain of *Ganoderma lucidum* QRS 5120 exhibited higher LC_50_ values, 1.650 mg/ml and 2.648 mg/ml, respectively, compared to ENS-BG (0.920 mg/ml). The difference in the LC_50_ values between the three extracts (MB, EPS, and ENS) was possibly related to the fact that they were extracted from different parts of the *Ganoderma lucidum* mycelial culture and using different extraction procedures, which could have resulted in different amounts or compositions of compounds (Ma et al., 2018; Taufek et al., 2020; Wan-Mohtar et al., 2021).

Taufek et al. (2020) explained that EPS derived from GL mycelium demonstrated a wide range of bioactivities, including immunostimulant and antitumorigenic effects (Ahmadifar et al., 2019), that are higher than those derived from the fruiting bodies (Kozarski et al., 2019). Meanwhile, the LC_50_ value of ENS was lower than that of EPS due to its different mycelial extraction methodology; EPS is extracted directly from the liquid culture of GL, whereas ENS is derived from the internal part of dried fungal mycelium through a series of physicochemical extractions (Abdullah et al., 2020).

Dulay et al. (2012) found that tail malformation indicated by the bent tail and S-shaped tail could be observed in zebrafish embryos exposed to a 1% extract (10 mg/ml) of *G. lucidum* fruiting body. Moreover, the same morphological abnormality was observed in all embryos treated with a 5% extract. This finding indicated that the safest concentration limit for the *G. lucidum* fruiting body extract was <1%, and the value is less toxic compared to the current finding for ENS-BG. Nonetheless, this study used an extract from a submerged culture, which has a greater potential for increasing the mycelial production in a smaller space and less time, with fewer chances of contamination, compared to polysaccharides yielded from *G. lucidum* fruiting bodies, which were lower in quantity and relatively expensive, and thus, the health-promoting and therapeutic benefits of *G. lucidum* will be limited (Ye et al., 2018).

Different methods were used in several studies to assess the toxicity of *G. lucidum* extract. A previous study by Handrianto and Wardani, (2019) reported the toxic effect of methanol extract from *G. lucidum* on brine shrimp with an LC_50_ value of 7,652 ppm and suggested using other extraction methods. However, according to Sharif et al. (2017), both aqueous and methanolic extracts are safe for human erythrocytes and should be further explored as food supplements and chemo-preventive drugs in future.

As previously reported, EPS from *G. lucidum* displayed a non-toxic effect on healthy human prostate cells (PN2TA) (Wan-Mohtar et al., 2016b) and normal human lung cells (WRL68) (Chung et al., 2001), while the study by Vitak et al. (2015) found that the mycelial biomass powder was safe when tested using Wistar outbred white male rats. A recent study by Taufek et al. (2020) reported that both non-toxic mycelial biomass and EPS from the same strain as current research. However, none of these published studies reported the toxicity assessment of the ENS extract from *G. lucidum*. Hence, this study clarified that ENS-BG is safe by using the zebrafish embryo model and completed the toxicity data on MB-EPS-ENS consortia for this *Ganoderma lucidum* QRS 5120.

### 3.3 *In vivo* antihyperglycemic activity of EPS-BG and ENS-BG on adult STZ-induced diabetic zebrafish

#### 3.3.1 Development of the STZ-induced diabetes zebrafish model

The zebrafish model of diabetes development consisted of two key procedures, including being overfed before the injection of STZ. This approach was inspired by the high-fat diet-fed, streptozotocin-treated rat model of type 2 diabetes (Skovsø, 2014). For STZ injection in zebrafish, the method was carried out according to the method developed by Intine et al. (2013) and Abbas et al. (2017) with few modifications, as the preliminary result showed an almost 100% mortality rate after the fourth dose of STZ injection. The same result was reported by Benchoula et al. (2019b) with an 80% mortality rate using the same method, which was attributed to stress generated by the long duration of the experiment and toxicity caused by the enormous volume of STZ injected into the fish organs, such as the liver. Hence, in this study, only three doses of 0.3% STZ were administered on days 1, 3, and 5, with one dose administered on each of the 3 days mentioned.

**FIGURE 11:**
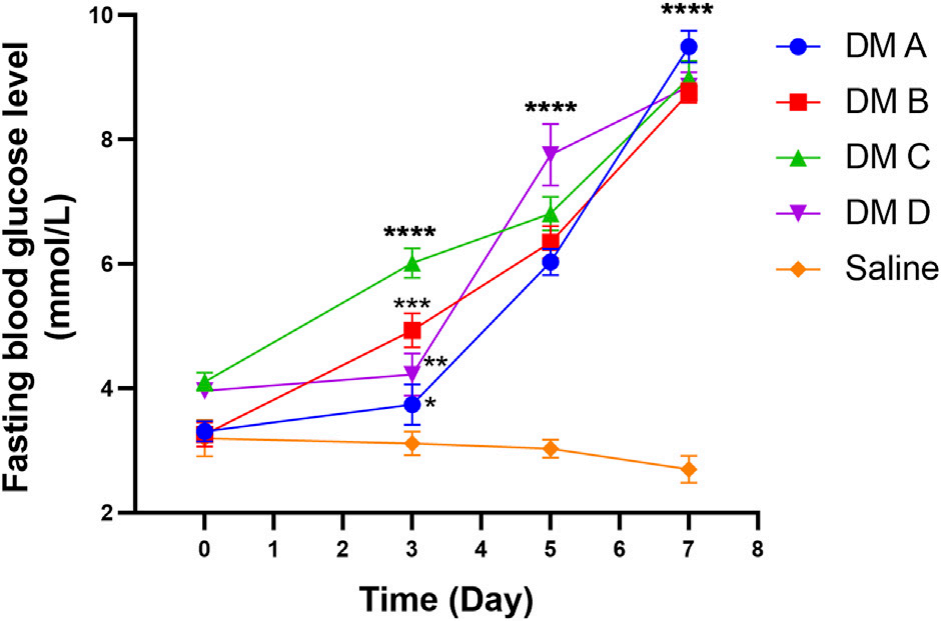
Fasting blood glucose level of zebrafish analyzed at different times after the injection of STZ (0.35 mg/g). Data are expressed as mean ± SEM, *n* = 15. One-way analysis of variance (ANOVA) was used to carry out the significant differences with a post-hoc test using Dunnett’s Multiple Comparison. The significant difference was considered at *p<0.05, **p < 0.01, ***p < 0.001, and ****p < 0.0001 between the means of the STZ-induced group compared to zebrafish induced with saline (control).

Figure 11 shows the fasting blood glucose level (BGL) of zebrafish at different time points of the experiment. The reading for normal fasting BGL is <6.1 mmol/L, and a reading of ≥7.0 mmol/L is considered diabetic. On day 0, all groups exhibited the same starting BGL as indicated by the insignificant difference in the reading compared to the normal group (*p* > 0.05). A notable increasing trend in fasting BGL of STZ-induced zebrafish was observed after the first dosage injection when compared to the control group that was treated with saline. Fasting BGL was found to be high following the first and second doses of STZ compared to fasting BGL before STZ administration. However, the fasting BGL remains within the normal range, which may be due to the ability of the zebrafish to recover and the high regeneration of the β-cells (Intine et al., 2013; Benchoula et al., 2019b). Nevertheless, on day 7 which was 2 days after the 3rd dose of STZ injection, all groups that received STZ showed a highly significant difference in fasting BGL as compared to the control group injected with saline (*p* < 0.0001). There was no significant difference seen in fasting BGL for the group injected with saline as compared to the BGL reading before saline injection, which indicates that there was no effect of saline and the injection procedure on zebrafish BGL.

#### 3.3.2 Oral sucrose tolerance test

All the treatment groups, including control groups, proceeded to oral sucrose tolerance tests (OSTT). This test may indicate that the control of postprandial glucose levels shown by EPS-BG and ENS-BG might be mediated by the regulation of glucose uptake from the intestinal lumen through the inhibition of complex carbohydrate digestion (OSTT). The absence of the postprandial peak after loading sucrose can be associated with the inhibition of alpha-glucosidase or the inhibition of sodium-glucose cotransporter type 1 SGLT1 (Valdes et al., 2019).

**FIGURE 12:**
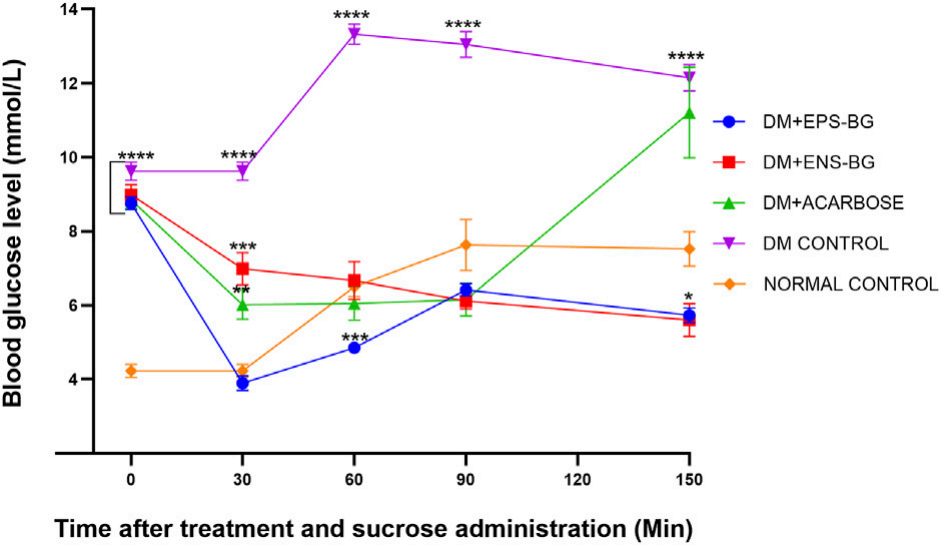
Effect of EPS-BG, ENS-BG, and acarbose on the oral sucrose tolerance test. Treatment was orally administered to zebrafish 30 min before sucrose loading. The postprandial blood glucose level was observed for 2 h. Data are expressed as mean ± SEM, *n* = 15. One-way analysis of variance (ANOVA) was used to carry out the significant differences with a post-hoc test using Dunnett’s Multiple Comparison. The significant difference was considered at *p ˂ 0.05, **p < 0.01, ***p < 0.001, and ****p < 0.0001 between the means of groups compared to the control normal healthy group.

Figure 12 shows the blood glucose level of zebrafish after oral administration of sucrose for 2 hours. Thirty minutes before sucrose delivery, the zebrafish were treated with acarbose, EPS-BG, ENS-BG, and saline solution for the control group. DM zebrafish groups started with the same baseline of the BGL, which is very significant compared to the normal control group (*p* < 0.0001). To start the experiment at 0 min, the zebrafish were treated with EPS-BG, ENS-BG, acarbose (a positive control), and saline for the control group of healthy and diabetic zebrafish. After 30 min of treatment, the reading of BGL was significantly decreased for the group treated with EPS-BG (8.750 ± 0.170 mmol/L to 3.892 ± 0.197 mmol/L), ENS-BG (8.983 ± 0.2752 mmol/L to 6.983 ± 0.43 mmol/L), and acarbose (8.850 ± 0.231 mmol/L to 6.017 ± 0.393 mmol/L).

After 30 min of sucrose testing, the highest peak of BGL was recorded in the DM control group (13.325 0.273 mmol/L). This result was constant with the previous study by Eames et al. (2010), where the peak blood glucose was observed 30 min after sucrose loading. The BGL of the diabetic control group was compared to the treatment group and the BGL of the normal healthy group. There were extremely significant differences between the DM control group and the group of DM zebrafish treated with EPS-BG, ENS-BG, and acarbose. These OSTT data demonstrated that EPS-BG, ENS-BG, and acarbose significantly lowered the blood glucose levels of DM zebrafish with insignificant differences compared to the control normal healthy group. In this regard, a putative action on glucosidase enzymes and/or SGLT2 may be a contributing factor to the mechanism by which the *G. lucidum* extract decreased postprandial hyperglycemia (Calzada et al., 2019).

**FIGURE 13:**
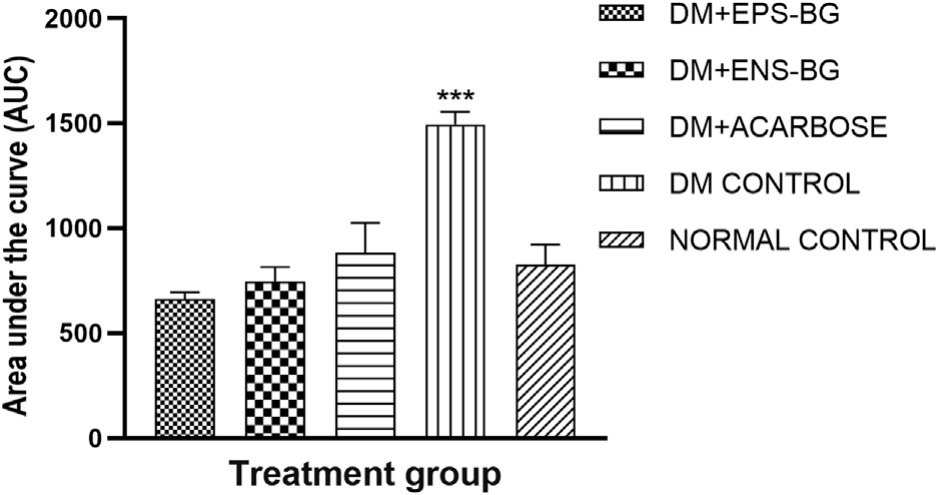
Area under the curve (AUC) of the zebrafish blood glucose level after oral sucrose administration. Data are expressed as mean ± SEM. One-way analysis of variance (ANOVA) was used to carry out the significant differences with a post-hoc test using Dunnett’s Multiple Comparison. The significant difference was considered at ***p < 0.001 between the means of groups compared to the control normal healthy group.

The AUC, which is an indicator of whole glucose excursion after glucose loading, has been widely used to calculate the glycemic index and assess the efficacy of postprandial hyperglycemia treatments (Sakaguchi et al., 2016). The AUC for the treatment of the control groups is shown in Figure 13. This research demonstrated the zebrafish’s efficacy in controlling the BGL after sucrose delivery with determined treatments (EPS-BG, ENS-BG, and acarbose). Refer to Figure 12, which explains the BGL reading of zebrafish following sucrose administration; the zebrafish groups treated with EPS-BG and ENS-BG reduced the BGL and inhibited the sudden rise of BGL for 2 hours of OSTT compared to the control DM group. The data for EPS-BG and ENS-BG shown in Figure 13 were comparable and not significant to the normal control group and the group treated with a standard drug, acarbose. The overall effects of the BGL after sucrose loading measured as the AUC shows that EPS-BG (*p* = 0.5024) and ENS-BG (*p* = 0.9248) were not significant compared to the normal control group. The non-significant result of EPS-BG and ENS-BG, when compared to the normal control group, indicates that both treatments have a good effect on blood glucose regulation in DM zebrafish after sucrose administration. Furthermore, when compared to the normal control group, the AUC for the DM control group was extremely significant, with a *p*-value <0.0001. Hence, it can be suggested that EPS-BG and ENS-BG extracted from GL strain QRS 5120 could regulate postprandial hyperglycemia to normal BGL comparable to those observed in the normal control group and did not cause hypoglycemia.

## 4 Conclusion

To conclude, these works remarkably present that both EPS-BG and ENS-BG demonstrated strong inhibition of alpha-glucosidase activity similar to the clinically approved alpha-glucosidase inhibitor, acarbose. ZFET revealed that ENS-BG is non-toxic and safe to be used as a potential natural alpha-glucosidase inhibitor. *In vivo* findings suggest that EPS-BG and ENS-BG had a noteworthy effect on lowering the blood glucose level in induced diabetic adult zebrafish and significantly lowering peak blood glucose and the area under the curve (AUC) in OSTT.

## Data Availability

The original contributions presented in the study are included in the article/Supplementary Material; further inquiries can be directed to the corresponding author.

## References

[B1] Abbas Q. Hassan M. Raza H. Kim S. J. Chung K. W. Kim G. H. (2017). *In vitro*, *in vivo* and *in silico* anti-hyperglycemic inhibition by sinigrin. Asian Pac. J. Trop. Med. 10, 372–379. 10.1016/j.apjtm.2017.03.019 28552107

[B2] Abdullah N. R. Sharif F. Azizan N. H. Hafidz I. F. M. Supramani S. Usuldin S. R. A. (2020). Pellet diameter of ganoderma lucidum in a repeated-batch fermentation for the trio total production of biomass-exopolysaccharide-endopolysaccharide and its anti-oral cancer beta-glucan response. AIMS Microbiol. 6, 379–400. 10.3934/microbiol.2020023 33364534 PMC7755588

[B3] Ahmad R. Riaz M. Khan A. Aljamea A. Algheryafi M. Sewaket D. (2021). *Ganoderma lucidum* (Reishi) an edible mushroom; a comprehensive and critical review of its nutritional, cosmeceutical, mycochemical, pharmacological, clinical, and toxicological properties. Phytotherapy Res. 35, 6030–6062. 10.1002/PTR.7215 34411377

[B4] Ahmad Usuldin S. R. Mahmud N. Ilham Z. Khairul Ikram N. K. Ahmad R. Wan-Mohtar W. A. A. Q. I. (2020). In-depth spectral characterization of antioxidative (1, 3)-β-D-glucan from the mycelium of an identified tiger milk mushroom *Lignosus rhinocerus* strain ABI in a stirred-tank bioreactor. Biocatal. Agric. Biotechnol. 23, 101455. 10.1016/j.bcab.2019.101455

[B5] Ahmadifar E. Dawood M. A. O. Moghadam M. S. Sheikhzadeh N. Hoseinifar S. H. Musthafa M. S. (2019). Modulation of immune parameters and antioxidant defense in zebrafish (*Danio rerio*) using dietary apple cider vinegar. Aquaculture 513, 734412. 10.1016/J.AQUACULTURE.2019.734412

[B6] Akmal M. Wadhwa R. (2021). Alpha glucosidase inhibitors. StatPearls Publishing, Tampa, Florida, United States. 32496728

[B7] Baby S. Johnson A. J. Govindan B. (2015). Secondary metabolites from ganoderma. Phytochemistry 114, 66–101. 10.1016/j.phytochem.2015.03.010 25975187

[B8] Bakkers J. (2011). Zebrafish as a model to study cardiac development and human cardiac disease. Cardiovasc. Res. 91, 279–288. 10.1093/cvr/cvr098 21602174 PMC3125074

[B9] Bauer B. Mally A. Liedtke D. (2021). Zebrafish embryos and larvae as alternative animal models for toxicity testing. Int. J. Mol. Sci. 22, 13417. 10.3390/ijms222413417 34948215 PMC8707050

[B10] Benchoula K. KhatiB A. Jaffar A. Udin Ahmed Q. Mohd Azizi Wan Sulaiman W. Abd Wahab R. (2019a). The promise of zebrafish as a model of metabolic syndrome. Jpn. Assoc. Laboratory Animal Sci. 68, 407–416. 10.1538/expanim.18-0168 PMC6842808 31118344

[B11] Benchoula K. Khatib A. Quzwain F. M. C. Che Mohamad C. A. Wan Sulaiman W. M. A. Wahab R. A. (2019b). Optimization of hyperglycemic induction in zebrafish and evaluation of its blood glucose level and metabolite fingerprint treated with *Psychotria malayana* jack leaf extract. Molecules 24, 1506. 10.3390/molecules24081506 PMC6515116 30999617

[B12] Calzada F. Valdes M. Garcia-Hernandez N. Velázquez C. Barbosa E. Bustos-Brito C. (2019). Antihyperglycemic activity of the leaves from *Annona diversifolia* safford. And farnesol on normal and alloxan-induced diabetic mice. Pharmacogn. Mag. 15, S38–S46. 10.4103/pm.pm

[B13] Chaudhury A. Duvoor C. Reddy Dendi V. S. Kraleti S. Chada A. Ravilla R. (2017). Clinical review of antidiabetic drugs: Implications for type 2 diabetes mellitus management. Front. Endocrinol. 8, 6. 10.3389/fendo.2017.00006 PMC5256065 28167928

[B14] Chen B. Tian J. Zhang J. Wang K. Liu L. Yang B. (2017). Triterpenes and meroterpenes from *Ganoderma lucidum* with inhibitory activity against HMGs reductase, aldose reductase and α-glucosidase. Fitoterapia 120, 6–16. 10.1016/j.fitote.2017.05.005 28527898

[B15] Chen S.-D. Yong T. Zhang Y. Hu H.-P. Xie Y.-Z. (2019). Inhibitory effect of five ganoderma species (agaricomycetes) against key digestive enzymes related to type 2 diabetes mellitus. Int. J. Med. Mushrooms 21, 703–711. 10.1615/IntJMedMushrooms.v21.i7.70 31679304

[B16] Chung W. T. Lee S. H. Kim J. D. Park Y. S. Hwang B. Lee S. Y. (2001). Effect of mycelial culture broth of Ganoderma lucidum on the growth characteristics of human cell lines. J. Biosci. Bioeng. 92, 550–555. 10.1016/S1389-1723(01)80314-5 16233144

[B17] Du Q. Cao Y. Liu C. (2021). “Lingzhi, an overview,” in The lingzhi mushroom genome. Editor Liu C. (Cham: Springer International Publishing), 1–25. 10.1007/978-3-030-75710-6_1

[B18] Dulay R. M. R. Kalaw S. P. Reyes R. G. Alfonso N. F. Eguchi F. (2012). Teratogenic and toxic effects of Lingzhi or Reishi medicinal mushroom, *Ganoderma lucidum* (W.Curt.:Fr.) P. Karst. (Higher basidiomycetes), on zebrafish embryo as model. Int. J. Med. Mushrooms 14, 507–512. 10.1615/IntJMedMushr.v14.i5.90 23510220

[B19] Eames S. C. Philipson L. H. Prince V. E. Kinkel M. D. (2010). Blood sugar measurement in zebrafish reveals dynamics of glucose homeostasis. Zebrafish 7, 205–213. 10.1089/zeb.2009.0640 20515318 PMC2882991

[B20] Handrianto P. Wardani R. K. (2019). Uji toksisitas ekstrak metanol lingzhi (*ganoderma lucidum*) dengan metode brine shrimp lethality test (BSLT). J. Res. Technol. 5, 3459378. 10.5281/ZENODO.3459378

[B21] Heckler K. Kroll J. (2017). Zebrafish as a model for the study of microvascular complications of diabetes and their mechanisms. Int. J. Mol. Sci. 18, 2002–2009. 10.3390/ijms18092002 PMC5618651 28925940

[B22] Intine R. V. Olsen A. S. Sarras M. P. (2013). A zebrafish model of diabetes mellitus and metabolic memory. J. Vis. Exp. 72, e50232. 1–7. 10.3791/50232 PMC3622110 23485929

[B23] Ji Y. Liu D. Zhao J. Zhao J. Li H. Li L. (2021). *In vitro* and *in vivo* inhibitory effect of anthocyanin-rich bilberry extract on α-glucosidase and α-amylase. LWT - Food Sci. Technol. 145, 111484. 10.1016/j.lwt.2021.111484

[B24] Kazeem M. I. Adamson J. O. Ogunwande I. A. (2013). Modes of inhibition of α-amylase and α-glucosidase by aqueous extract of *Morinda lucida* benth leaf. BioMed Res. Int. 2013, 1–6. 10.1155/2013/527570 PMC3884628 24455701

[B25] Kinkel M. D. Eames S. C. Philipson L. H. Prince V. E. (2010). Intraperitoneal injection into adult zebrafish. J. Vis. Exp. 42, 2126. 10.3791/2126 PMC3278329 20834219

[B26] Kozarski M. Klaus A. Jakovljević D. Todorović N. Imad Wan-Mohtar W. A. A. Q. Nikšić M. (2019). *Ganoderma lucidum* as a cosmeceutical: Antiradical potential and inhibitory effect on hyperpigmentation and skin extracellular matrix degradation enzymes. Arch. Biol. Sci. 71, 253–264. 10.2298/ABS181217007K

[B27] Krishnan J. Rohner N. (2019). Sweet fish: Fish models for the study of hyperglycemia and diabetes. J. Diabetes 11, 193–203. 10.1111/1753-0407.12860 30264455

[B28] Lankatillake C. Huynh T. Dias D. A. (2019). Understanding glycaemic control and current approaches for screening antidiabetic natural products from evidence-based medicinal plants. Plant Methods 15, 105–135. 10.1186/s13007-019-0487-8 31516543 PMC6731622

[B60] Liu M. Lan Y. Tian C. Zhu Y. Liu H. Wang W. (2018). The characterization, renoprotection and antioxidation of enzymatic and acidic exopolysaccharides from *Hypsizigus marmoreus* . Sci. Rep. 8 (1), 1–11. 10.1038/s41598-018-20440-y 29391516 PMC5794867

[B29] Luthra T. Agarwal R. Estari M. Adepally U. Sen S. (2017). A novel library of -arylketones as potential inhibitors of α-glucosidase: Their design, synthesis, *in vitro* and *in vivo* studies. Sci. Rep. 7, 13246–13313. 10.1038/s41598-017-13798-y 29038580 PMC5643545

[B30] Ma X. Ding Y. Wang Y. Xu X. (2018). A doxorubicin-induced cardiomyopathy model in adult zebrafish. J. Vis. Exp. 2018, 2–9. 10.3791/57567 PMC6101627 29939187

[B31] Mathews B. M. Gustafsson E. (2019). A Zebrafish model system for drug screening in diabetes. (Skövde, Sweden: University of Skövde). 40.

[B32] Matthews M. Varga Z. M. (2012). Anesthesia and euthanasia in zebrafish. ILAR J. 53, 192–204. 10.1093/ilar.53.2.192 23382350

[B33] Middel C. S. Hammes H.-P. P. Kroll J. (2021). Advancing diabetic retinopathy research: Analysis of the neurovascular unit in zebrafish. Cells 10, 1313. 10.3390/cells10061313 34070439 PMC8228394

[B34] Mohammed A. Gbonjubola V. A. Koorbanally N. A. Islam M. S. (2017). Inhibition of key enzymes linked to type 2 diabetes by compounds isolated from *Aframomum melegueta* fruit. Pharm. Biol. 55, 1010–1016. 10.1080/13880209.2017.1286358 28176546 PMC6130490

[B35] Nicolaus B. Kambourova M. Oner E. T. (2010). Exopolysaccharides from extremophiles: From fundamentals to biotechnology. Environ. Technol. 31, 1145–1158. 10.1080/09593330903552094 20718297

[B36] OECD (2013). OECD guidelines for the testing of chemicals, section 2. Test No. 236: Fish Embryo Acute Toxicity (FET) Test. Paris, France: OECD Publishing, 1–22. 10.1787/9789264203709-en

[B37] Paterson R. R. M. (2006). Ganoderma - a therapeutic fungal biofactory. Phytochemistry 67, 1985–2001. 10.1016/j.phytochem.2006.07.004 16905165

[B38] Poli A. Di Donato P. Abbamondi G. R. Nicolaus B. (2011). Synthesis, production, and biotechnological applications of exopolysaccharides and polyhydroxyalkanoates by Archaea. Archaea 2011, 693253. 10.1155/2011/693253 22007151 PMC3191746

[B58] Priatni S. Budiwati T. A. Ratnaningrum D. Kosasih W. Andryani R. Susanti H. (2016). Antidiabetic screening of some Indonesian marine cyanobacteria collection. Biodivers. 17 (2), 642–646. 10.13057/biodiv/d170236

[B39] Sakaguchi K. Takeda K. Maeda M. Ogawa W. Sato T. Okada S. (2016). Glucose area under the curve during oral glucose tolerance test as an index of glucose intolerance. Diabetol. Int. 7, 53–58. 10.1007/S13340-015-0212-4 30603243 PMC6214468

[B40] Salehpour A. Rezaei M. Khoradmehr A. Tahamtani Y. Tamadon A. (2021). Which hyperglycemic model of zebrafish (*Danio rerio*) suites my type 2 diabetes mellitus research? A scoring system for available methods. Front. Cell Dev. Biol. 9, 652061. 10.3389/fcell.2021.652061 33791308 PMC8005598

[B59] Sasikumar K. Kozhummal Vaikkath D. Devendra L. Nampoothiri K. M. (2017). An exopolysaccharide (EPS) from a *Lactobacillus plantarum* BR2 with potential benefits for making functional foods. Bioresour. Technol. 241, 1152–1156. 10.1016/j.biortech.2017.05.075 28579175

[B41] Satria D. Tamrakar S. Suhara H. Kaneko S. Shimizu K. (2019). Mass spectrometry-based untargeted metabolomics and α-glucosidase inhibitory activity of lingzhi (ganoderma lingzhi) during the developmental stages. Molecules 24, 2044. 10.3390/MOLECULES24112044 PMC6600326 31146329

[B42] Seweryn E. Ziała A. Gamian A. (2021). Health-promoting of polysaccharides extracted from *Ganoderma lucidum* . Nutrients 13, 2725. 10.3390/NU13082725 34444885 PMC8400705

[B43] Sharif S. Shahid M. Mushtaq M. Akram S. Rashid A. (2017). Wild mushrooms: A potential source of nutritional and antioxidant attributes with acceptable toxicity. Prev. Nutr. Food Sci. 22, 124–130. 10.3746/pnf.2017.22.2.124 28702429 PMC5503421

[B44] Skovsø S. (2014). Modeling type 2 diabetes in rats using high fat diet and streptozotocin. J. Diabetes Investig. 5, 349–358. 10.1111/JDI.12235 PMC4210077 25411593

[B45] Taufek N. M. Harith H. H. Abd Rahim M. H. Ilham Z. Rowan N. Wan-Mohtar W. A. A. Q. I. (2020). Performance of mycelial biomass and exopolysaccharide from Malaysian *Ganoderma lucidum* for the fungivore red hybrid Tilapia (*Oreochromis sp.*) in Zebrafish embryo. Aquac. Rep. 17, 100322. 10.1016/j.aqrep.2020.100322

[B46] Valdes M. Calzada F. Mendieta-Wejebe J. (2019). Structure-activity relationship study of acyclic terpenes in blood glucose levels: Potential α-Glucosidase and Sodium Glucose Cotransporter (SGLT-1) Inhibitors. Molecules 24, 4020. 10.3390/molecules24224020 PMC6891574 31698833

[B47] Vitak T. Y. Wasser S. P. Nevo E. Sybirna N. O. (2015). The effect of the medicinal mushrooms *Agaricus brasiliensis* and *Ganoderma lucidum* (higher basidiomycetes) on the erythron system in normal and streptozotocin-induced diabetic rats. Int. J. Med. Mushrooms 17, 277–286. 10.1615/INTJMEDMUSHROOMS.V17.I3.70 25954911

[B48] Wan Mohtar W. A. A. Q. I. Latif N. A. Harvey L. M. McNeil B. (2016a). Production of exopolysaccharide by *Ganoderma lucidum* in a repeated-batch fermentation. Biocatal. Agric. Biotechnol. 6, 91–101. 10.1016/j.bcab.2016.02.011

[B49] Wan-Mohtar W. A. A. Q. I. Ilham Z. Jamaludin A. A. Rowan N. (2021). Use of zebrafish embryo assay to evaluate toxicity and safety of bioreactor- grown exopolysaccharides and endopolysaccharides from European *Ganoderma applanatum* mycelium for future aquaculture applications. Int. J. Mol. Sci. 22, 1675. 10.3390/ijms22041675 33562361 PMC7914815

[B50] Wan-Mohtar W. A. A. Q. I. Young L. Abbott G. M. Clements C. Harvey L. M. McNeil B. (2016b). Antimicrobial properties and cytotoxicity of sulfated (1, 3)-β-D-glucan from the mycelium of the mushroom *Ganoderma lucidum* . J. Microbiol. Biotechnol. 26, 999–1010. 10.4014/jmb.1510.10018 26907757

[B51] Ye L. Liu S. Xie F. Zhao L. Wu X. (2018). Enhanced production of polysaccharides and triterpenoids in Ganoderma lucidum fruit bodies on induction with signal transduction during the fruiting stage. PLOS ONE 13, e0196287. 10.1371/JOURNAL.PONE.0196287 29694432 PMC5919040

[B52] Zaharudin N. Staerk D. Dragsted L. O. (2019). Inhibition of α-glucosidase activity by selected edible seaweeds and fucoxanthin. Food Chem. 270, 481–486. 10.1016/j.foodchem.2018.07.142 30174076

[B53] Zang L. Maddison L. A. Chen W. (2018). Zebrafish as a model for obesity and diabetes. Front. Cell Dev. Biol. 6, 91–13. 10.3389/fcell.2018.00091 30177968 PMC6110173

[B54] Zang L. Shimada Y. Nishimura N. (2017). Development of a novel zebrafish model for type 2 diabetes mellitus. Sci. Rep. 7, 1461. 10.1038/s41598-017-01432-w 28469250 PMC5431185

[B55] Zang L. Shimada Y. Nishimura Y. Tanaka T. Nishimura N. (2015). Repeated blood collection for blood tests in adult zebrafish. J. Vis. Exp. 1, e53272. 10.3791/53272 PMC4692578 26383512

[B56] Zhang X. Li G. Wu D. Yu Y. Hu N. Wang H. (2020). Emerging strategies for the activity assay and inhibitor screening of alpha-glucosidase. Food Funct. 11, 66–82. 10.1039/C9FO01590F 31844870

[B57] Zhou L. W. Cao Y. Wu S. H. Vlasák J. Li D. W. Li M. J. (2015). Global diversity of the *Ganoderma lucidum* complex (Ganodermataceae, Polyporales) inferred from morphology and multilocus phylogeny. Phytochemistry 114, 7–15. 10.1016/j.phytochem.2014.09.023 25453909

