# Supplementary figures and images for: Bioreactor-grown exo- and endo-β-glucan from Malaysian Ganoderma lucidum: An in vitro and in vivo study for potential antidiabetic treatment
Source: Front Bioeng Biotechnol. 2022 Aug 25;10:960320. doi: 10.3389/fbioe.2022.960320 (PMC9452895; doi:10.3389/fbioe.2022.960320)

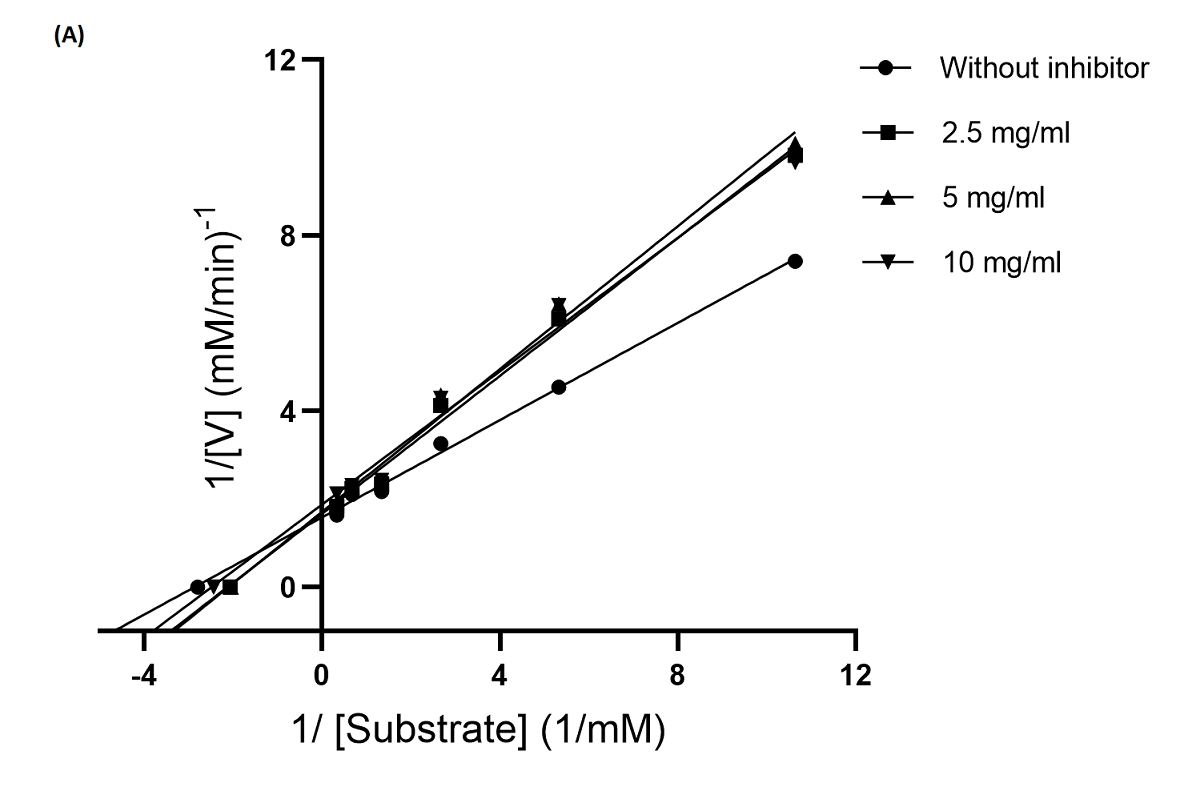

Supplement: Supplementary file 1 [file Image3.TIF]

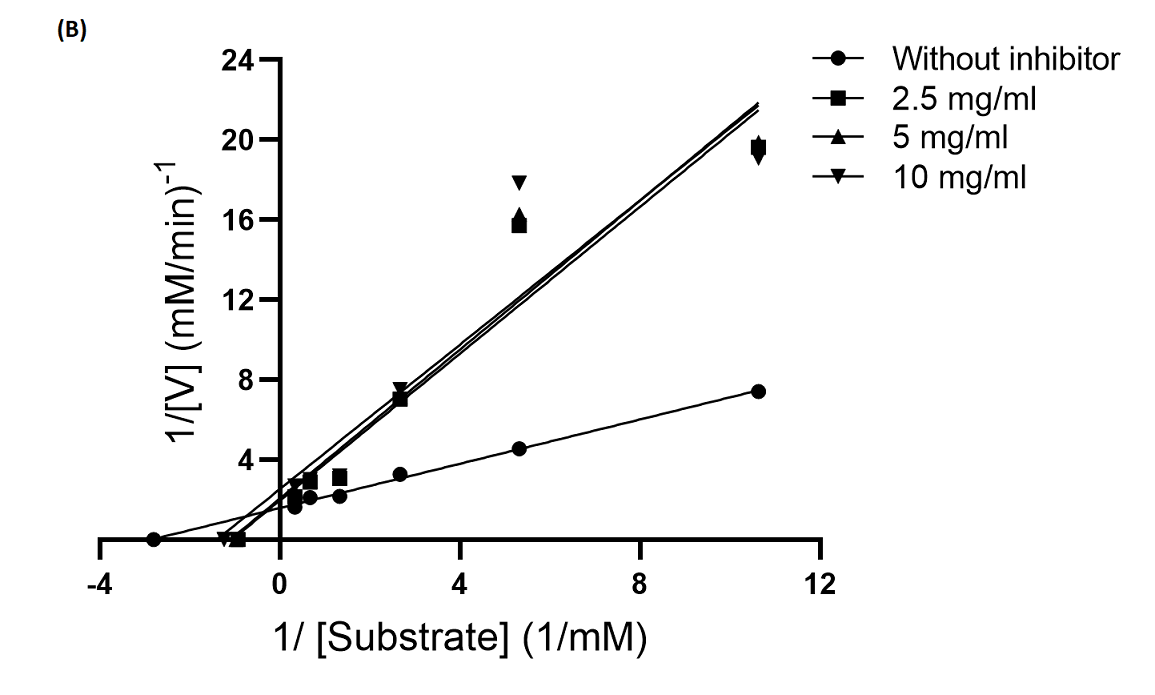

Supplement: Supplementary file 2 [file Image4.TIF]

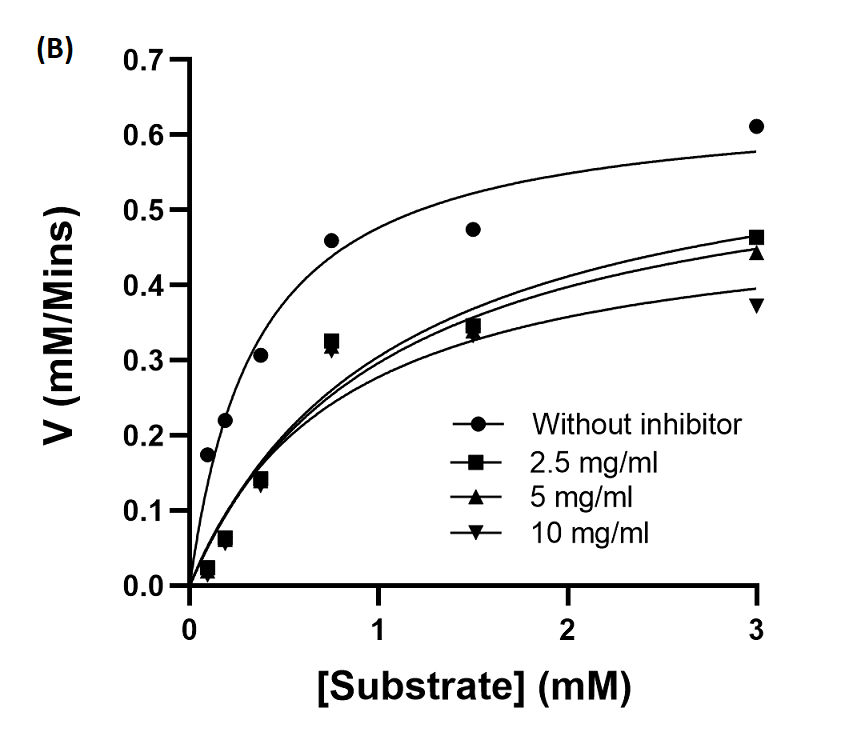

Supplement: Supplementary file 3 [file Image2.TIF]

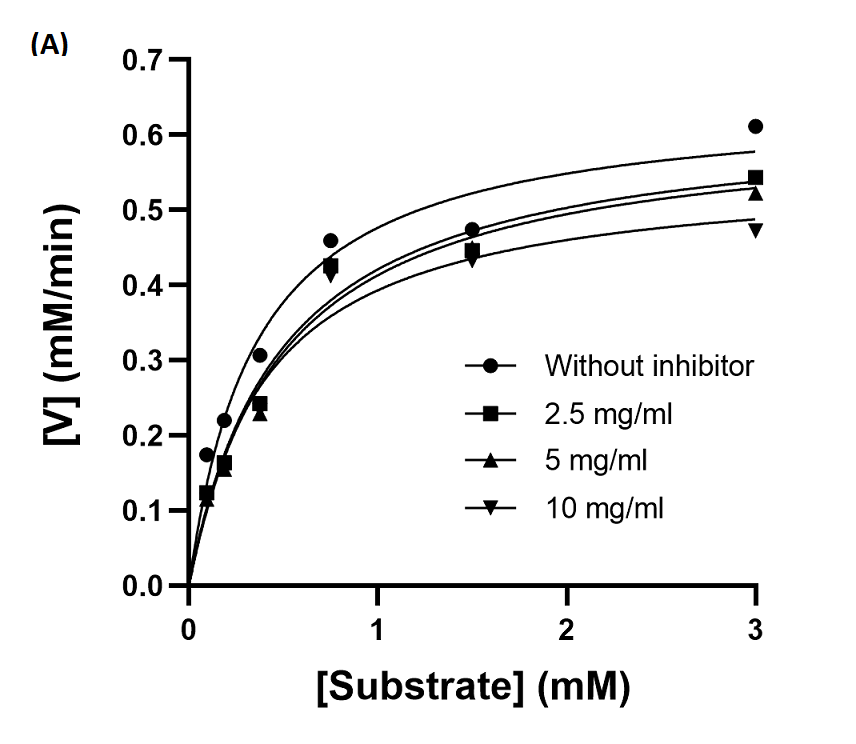

Supplement: Supplementary file 4 [file Image1.TIF]
