# Supplementary material for: Bioreactor-grown exo- and endo-β-glucan from Malaysian Ganoderma lucidum: An in vitro and in vivo study for potential antidiabetic treatment
Source: Front Bioeng Biotechnol. 2022 Aug 25;10:960320. doi: 10.3389/fbioe.2022.960320 (PMC9452895; doi:10.3389/fbioe.2022.960320)
Supplement: Supplementary file 5 [file DataSheet1.PDF]

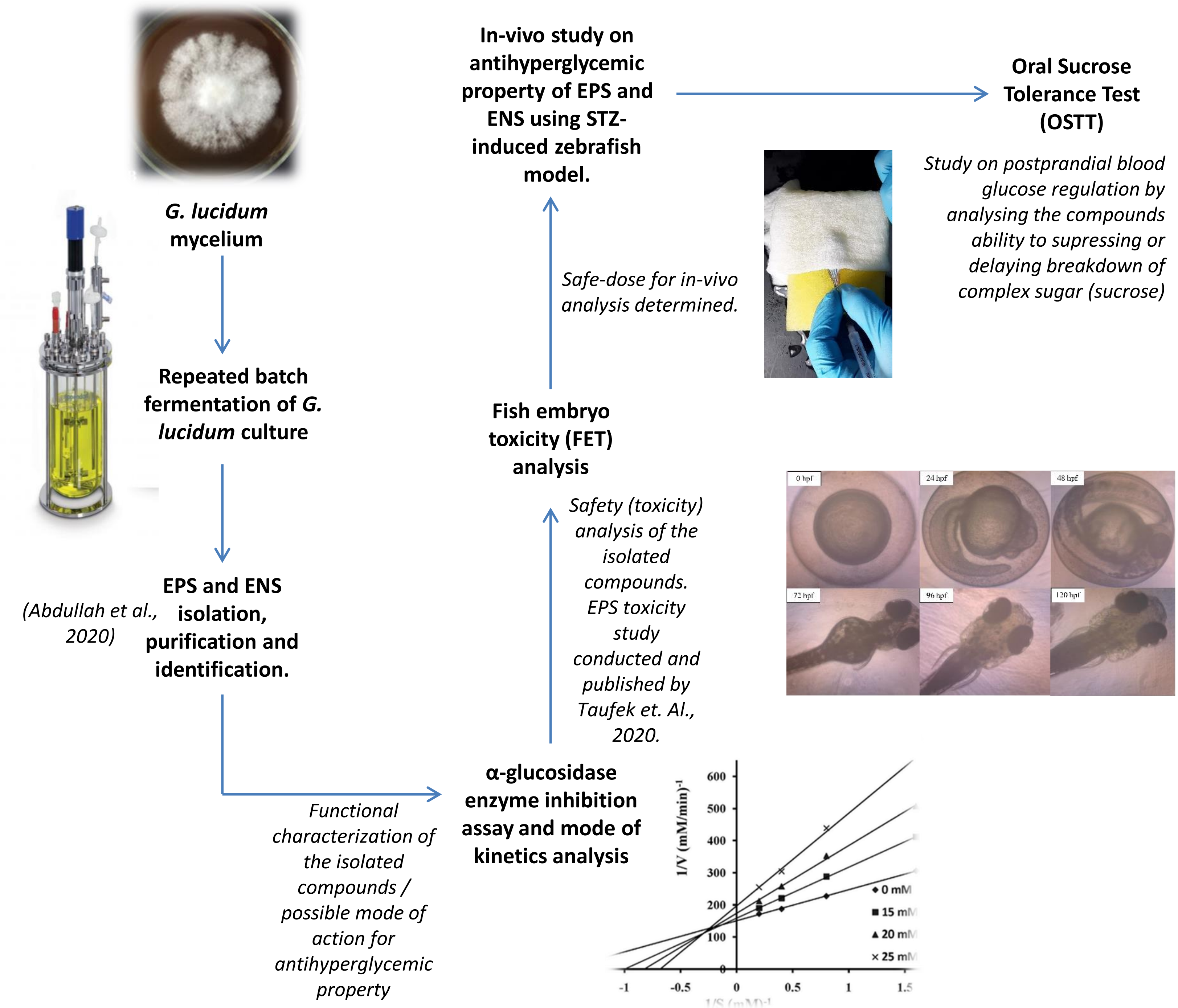
G. lucidum mycelium
Repeated batch fermentation of G. lucidum culture
EPS and ENS isolation, purification and identification.
(Abdullah et al., 2020)
Functional characterization of the isolated compounds / possible mode of action for antihyperglycemic property
α-glucosidase enzyme inhibition assay and mode of kinetics analysis
1/V (mM/min)-1
600
500
400
300
200
100
0
-1
-0.5
0
0.5
1
1.5
0 mM
15 mM
20 mM
25 mM
Safety (toxicity) analysis of the isolated compounds. EPS toxicity study conducted and published by Taufek et. Al., 2020.
Fish embryo toxicity (FET) analysis
0 hpf
24 hpf
48 hpf
72 hpf
96 hpf
120 hpf
Safe-dose for in-vivo analysis determined.
In-vivo study on antihyperglycemic property of EPS and ENS using STZ-induced zebrafish model.
Oral Sucrose Tolerance Test (OSTT)
Study on postprandial blood glucose regulation by analysing the compounds ability to supressing or delaying breakdown of complex sugar (sucrose)
